# Federated Anomaly Detection for IoT-Enabled EV Charging Cybersecurity Under Aggressive and Stealth Cyberattacks

**DOI:** 10.3390/s26175541

**Published:** 2026-08-31

**Authors:** Marran Al Qwaid, Gobbi Ramasamy, Md Sabbir Hossen

**Affiliations:** 1 Department of Computer Sciences, College of Computing and Information Technology, Shaqra University, Shaqra 11961, Saudi Arabia; marran@su.edu.sa; 2 Faculty of AI and Engineering, Multimedia University, Cyberjaya 63100, Malaysia; sabbir.cme@gmail.com

**Keywords:** electric vehicle charging infrastructure, IoT cybersecurity, federated learning, anomaly detection, LSTM autoencoder, privacy-preserving machine learning, stealth cyberattacks, cyber–physical systems

## Abstract

The rapid deployment of Internet of Things (IoT)-enabled electric vehicle (EV) charging infrastructures introduces cybersecurity challenges due to distributed operation, communication dependency, and exposure to malicious cyberattacks. Existing anomaly detection approaches mainly rely on centralized learning architectures that require raw charging data, creating privacy concerns, scalability limitations, and reduced robustness against stealth cyberattacks that mimic legitimate charging behavior. To address these issues, this paper proposes a privacy-preserving federated cybersecurity framework for IoT-based EV charging infrastructures using distributed anomaly detection. The framework integrates cybersecurity-aware feature engineering, aggressive and stealth cyberattack simulation, centralized anomaly detection, and federated learning without raw charging session sharing. A real-world EV charging dataset is utilized to model legitimate and malicious charging behavior using operational and engineered features, including charging ratio and power consistency. Experimental evaluation compares Isolation Forest, centralized LSTM autoencoder, and federated LSTM anomaly detection under aggressive and stealth cyberattack scenarios. The results show that aggressive cyberattacks are more easily separable, where Isolation Forest achieves an attack F1-score of 0.85 and AUC of 0.86, while stealth cyberattacks reduce detection performance, lowering the F1-score to 0.61. The centralized LSTM autoencoder demonstrates superior detection performance, whereas the federated LSTM preserves privacy and achieves competitive results with 0.86 accuracy, 0.67 attack F1-score, and 0.81 AUC without transmitting raw charging data. These findings demonstrate federated anomaly detection as a promising privacy-preserving solution for secure EV charging infrastructures.

## 1. Introduction

The rapid adoption of electric vehicles (EVs) has accelerated the deployment of smart charging infrastructures to support transportation electrification [1]. Modern EV charging systems increasingly rely on Internet of Things (IoT)-enabled communication, cloud connectivity, intelligent scheduling, and real-time monitoring to improve charging efficiency and operational flexibility [2]. Through IoT integration, charging stations exchange operational information and communicate with centralized platforms for supervision and analytics [3]. However, these capabilities also introduce cybersecurity vulnerabilities due to communication dependency, distributed environments, and expanded attack surfaces [4].

IoT-enabled EV charging systems are increasingly exposed to cyber threats, including false data injection, charging manipulation, session spoofing, unauthorized charging, and malicious control actions [5]. Such attacks may disrupt charging operations, distort measurements, and reduce operational trustworthiness [6]. Of particular concern are stealth cyberattacks, which mimic legitimate charging behavior and remain difficult to detect using conventional anomaly detection methods [7]. Their subtle perturbations substantially increase detection complexity in realistic adversarial environments [8].

Conventional cybersecurity frameworks primarily rely on centralized anomaly detection, where raw charging data are transmitted to central servers for training and attack analysis [9]. Although effective, centralized approaches introduce privacy risks, communication overhead, and scalability limitations in geographically distributed charging infrastructures [10,11,12,13].

Federated learning has emerged as a privacy-preserving distributed intelligence paradigm for IoT-enabled cyber–physical systems [14]. By exchanging model parameters instead of raw data, federated learning improves privacy preservation and collaborative intelligence across distributed infrastructures [15]. However, its application to cybersecurity-aware anomaly detection in EV charging systems remains limited, particularly under realistic stealth cyberattacks [16].

To address these limitations, this paper proposes a privacy-preserving federated cybersecurity framework integrating feature engineering, cyberattack simulation, centralized and federated anomaly detection, and federated LSTM-based learning for secure EV charging operations.

## 2. Related Work

The rapid expansion of Internet of Things (IoT)-enabled electric vehicle (EV) charging infrastructures has increased research interest in smart charging, cybersecurity, and anomaly detection [17]. Existing studies have explored charging optimization, intrusion detection, cyberattack resilience, and distributed intelligence for securing cyber–physical charging systems [18]. However, important gaps remain in privacy-preserving cybersecurity and realistic adversarial threat modeling for distributed EV charging environments.

Early research mainly focused on charging optimization, including scheduling, load balancing, demand forecasting, and smart grid integration, with limited attention to cybersecurity threats and malicious charging manipulation. As IoT-enabled charging systems adopted remote communication and cloud monitoring, cybersecurity emerged as a critical challenge [19]. Several studies investigated anomaly detection using statistical and machine learning methods, including clustering, decision trees, supporting vector machines, and reconstruction-based approaches [20]. Although effective, most frameworks rely on centralized learning architectures, introducing privacy concerns, communication overhead, scalability limitations, and single-point failure risks.

Deep learning approaches have demonstrated improved anomaly detection performance by modeling nonlinear and temporal charging behavior [21]. Recurrent neural networks, long short-term memory (LSTM), and autoencoder-based methods have shown effectiveness in detecting abnormal charging behavior through reconstruction error analysis [22]. However, these approaches remain largely centralized and often assume highly separable attacks, limiting applicability in realistic adversarial environments.

Federated learning has emerged as a privacy-preserving machine learning paradigm for distributed cyber–physical systems [23], enabling decentralized training through parameter exchange instead of raw data sharing [24]. Recent studies have applied federated learning to IoT and cybersecurity applications to improve privacy and collaborative intelligence [25]. Nevertheless, federated cybersecurity research for EV charging systems, particularly under stealth cyberattacks, remains limited [26].

Motivated by these gaps, this study proposes a privacy-preserving federated cybersecurity framework integrating charging-aware feature engineering, stealth and aggressive cyberattack modeling, and federated LSTM-based anomaly detection for secure EV charging infrastructures. Table 1 summarizes representative studies on EV charging cybersecurity, anomaly detection, IoT intrusion detection, and federated learning, highlighting the key limitations and research gap addressed by the proposed framework.

## 3. Methodology

### 3.1. Dataset Description

The experimental dataset consists of real-world electric vehicle (EV) charging session records collected from an Internet of Things (IoT)-enabled charging infrastructure. Each charging record contains operational information including charging timestamps, energy consumption, charging duration, average charging power, charging-station identifiers, and carbon-reduction metrics. The dataset contains 1929 charging sessions with 11 operational attributes representing heterogeneous charging behavior. The dataset was used as the legitimate baseline for cybersecurity evaluation because it does not contain verified real-world cyberattack labels; therefore, aggressive and stealth attack samples were generated through controlled perturbations of legitimate charging sessions. Before model development, duplicate sessions, incomplete observations, and missing values were handled to improve data consistency and maintain dataset integrity. Charging timestamps were converted into datetime format to derive temporal characteristics, including charging hour, weekday, and monthly activity, while charging duration was converted into numerical hours and numerical variables were standardized using z-score normalization. For federated learning, the processed charging records were distributed across four clients representing heterogeneous charging environments under non-IID conditions, with each client retaining its localized charging data for local LSTM autoencoder training. The experimental dataset was subsequently divided using an 80:20 stratified train–test split. Because this partition was performed at the session level rather than using a strict chronological or station-held-out protocol, sessions from the same charging environment and temporally adjacent operating periods may occur in both the training and testing subsets. Accordingly, the reported results represent session-level detection performance and should not be interpreted as a strict assessment of generalization to entirely unseen charging stations, users, or future time periods.

### 3.2. Data Preprocessing

Raw EV charging records were preprocessed to improve consistency and anomaly detection suitability. Duplicate sessions, incomplete observations, and missing values were handled to maintain dataset integrity. Charging timestamps were converted into datetime format to extract temporal features, including charging hour, weekday, and monthly activity. Charging duration was converted into numerical hour-based form, while numerical features were standardized using z-score normalization:

(1) $$x_{i}^{\prime }=\frac{x_{i}-\mu }{\sigma }\;$$

where $x_{i}$ denotes the original feature, μ is the feature mean, and σ is the standard deviation. Standardization improved numerical stability and reduced feature-scale variability for anomaly detection, deep learning, and federated learning experiments.

### 3.3. Proposed Federated Cybersecurity Framework

A privacy-preserving federated cybersecurity framework is proposed for IoT-based EV charging infrastructures using distributed anomaly detection.

**Figure 1:**
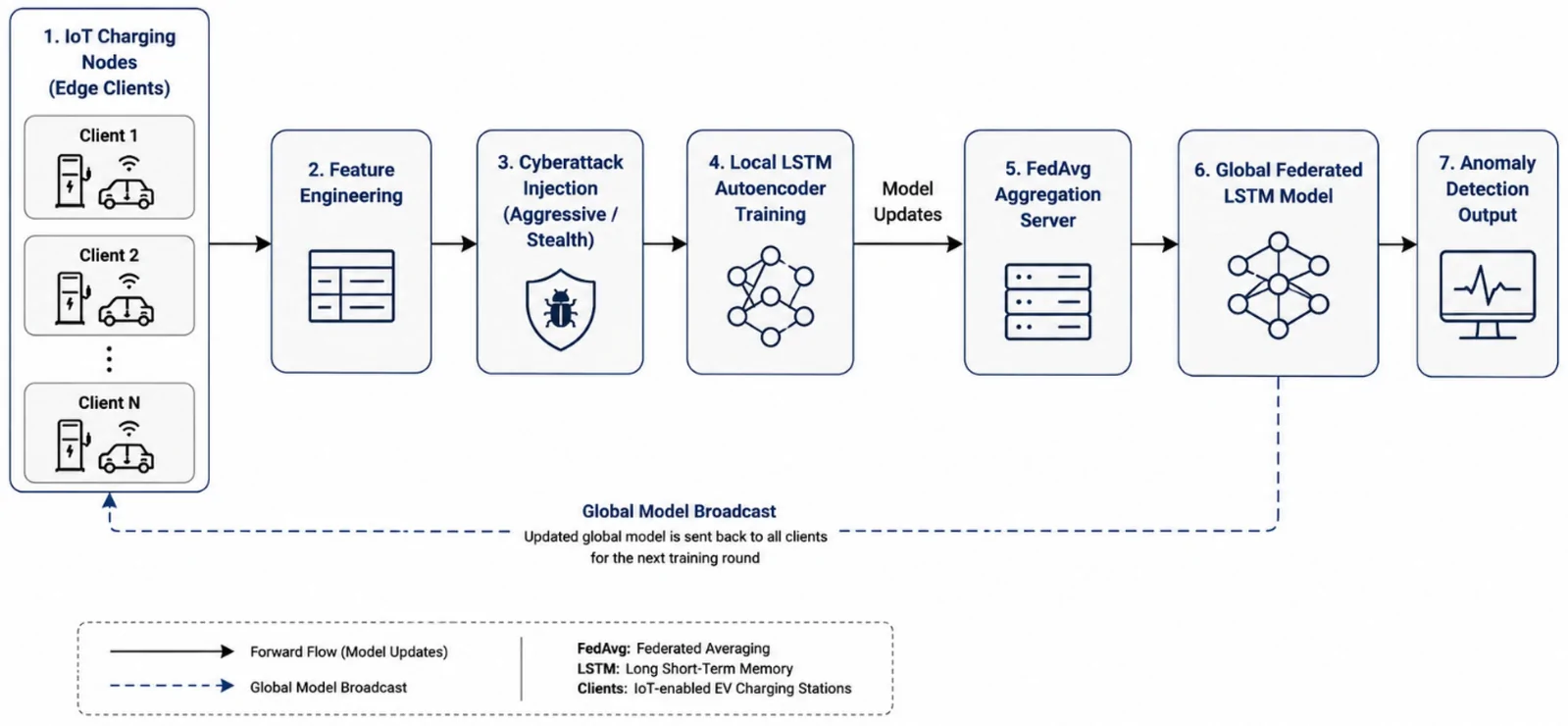
Proposed federated cybersecurity framework for IoT-based EV charging infrastructure using distributed anomaly detection, illustrating charging data acquisition, cyberattack injection, anomaly detection, and privacy-preserving federated learning.

As illustrated in Figure 1, the framework integrates charging data acquisition, preprocessing, cybersecurity-aware feature engineering, cyberattack simulation, anomaly detection, and federated learning. Charging characteristics are extracted and enhanced using engineered features, including charging ratio and power consistency, to improve separability between legitimate and malicious charging sessions.

To evaluate robustness, aggressive and stealth cyberattacks were simulated, where aggressive attacks introduce substantial charging perturbations and stealth attacks remain statistically similar to legitimate behavior. The anomaly detection stage combines Isolation Forest for statistical anomaly isolation and an LSTM autoencoder for temporal reconstruction-based detection. To preserve privacy, distributed charging nodes independently train local LSTM models using localized data while exchanging only model parameters through Federated Averaging (FedAvg). The framework enables privacy-preserving collaborative anomaly detection and distributed cybersecurity intelligence for EV charging environments under realistic cyberattack conditions.

### 3.4. Cybersecurity-Aware Feature Engineering

Cybersecurity-aware features were engineered from EV charging data to improve anomaly detection and capture charging inconsistencies indicative of cyberattacks. A charging ratio feature was defined to represent charging efficiency and behavioral consistency:

(2) $$CR_{i}=\frac{E_{i}}{T_{i}}\;$$

where $CR_{i}$ denotes the charging ratio of session i, $E_{i}$ is charging energy (kWh), and $T_{i}$ is charging duration (h). To further characterize charging consistency, power consistency was computed.

The power consistency formulation is expressed as follows:

(3) $$PC_{i}=\frac{P_{i}}{CR_{i}+\epsilon }\;$$

where $PC_{i}$ denotes power consistency, $P_{i}$ is average charging power, and $\epsilon =10^{-6}$ ensures numerical stability. Temporal features, including charging hour, weekday, and monthly activity, were additionally extracted to improve anomaly detection. Together, engineered and operational features provided behavioral representations for statistical, deep learning, and federated anomaly detection.

To avoid introducing artificial inconsistencies between the engineered and underlying operational variables, charging ratio and power consistency were treated as derived features rather than independently manipulated attack variables. During attack generation, the underlying charging variables were first modified according to the specified attack mechanism, after which the affected engineered features were recalculated from the resulting charging measurements. Consequently, the engineered features remain mathematically consistent with the corresponding energy, duration, and power values. This design also means that the engineered features may reflect changes introduced by the attack generator, which is considered when interpreting their contribution to anomaly separability.

### 3.5. Cyberattack Modeling

To evaluate anomaly detection robustness under adversarial conditions, two synthetic attack scenarios were generated from legitimate EV charging sessions: aggressive attacks representing highly disruptive manipulation and stealth attacks representing low-intensity charge manipulation. The underlying dataset contains 1929 legitimate charging sessions and no verified cyberattack records; therefore, the attack samples were generated through controlled perturbation of legitimate observations. A total of 269 sessions were manipulated for each attack scenario, corresponding to an attack injection proportion of approximately 14.0% relative to the original dataset.

Aggressive attack. For an original charging session i, let Ei denote charging energy (kWh), Di denote charging duration (h), and Pi denote average charging power (kW). The manipulated energy and duration were generated using multiplicative perturbation:

(4) Ei(a) =Ei (1+αi )Di(a) =Di (1+βi ) 

(5) Di(a)=Di (1+βi ) 

where αi and βi are attack-specific perturbation factors sampled from the aggressive-attack range. The resulting values were constrained to remain positive and within the operational bounds of the corresponding charging variables. The average charging power was then treated consistently with the manipulated energy and duration rather than independently assigning an unrelated value:

(6) $$P_{i}^{\left(a\right)}=\frac{E_{i}^{\left(a\right)}}{D_{i}^{\left(a\right)}}\;\;\;$$

This formulation preserves the fundamental physical relationship E≈PD and avoids introducing an algebraically inconsistent combination of energy, duration, and power. Derived charging features, including charging ratio and power consistency, were subsequently recalculated from the manipulated operational variables before model evaluation.

The engineered charging ratio and power consistency features were not independently assigned attack labels or perturbation values. Instead, they were recomputed after the underlying charging variables had been manipulated. This procedure preserves the mathematical relationship between the raw operational variables and their derived features and prevents the attack generator from creating internally contradictory feature combinations. However, because these features are derived from variables affected by the attack perturbations, their potential contribution to attack separability is acknowledged as an experimental limitation.

**Stealth attack.** The stealth scenario applied bounded low-intensity perturbations to charging behavior while maintaining similarity to legitimate sessions. For the implemented ±10% perturbation condition,

(7) Ei(s)=Ei (1+ϵE,i ),

(8) Di(s)=Di (1+ϵD,i ) 

(9) where ϵE,i,ϵD,i ∼U(−0.10,0.10).

The corresponding average power was recalculated from the perturbed energy and duration:

(10) $$P_{i}^{\left(s\right)}=\frac{E_{i}^{\left(s\right)}}{D_{i}^{\left(s\right)}}\;$$

The perturbed duration was constrained to remain positive, and the resulting charging variables were checked against the observed operational ranges of the source dataset. Temporal variables were retained from the corresponding legitimate charging session unless explicitly manipulated by the attack scenario. Because SOC measurements were not available in the source dataset, SOC was not directly modeled or perturbed in the present study. Consequently, the physical consistency constraints considered in the synthetic attacks were based on the available energy, duration, power, and timestamp variables.

Cybersecurity-aware derived features were recomputed after attack injection rather than independently perturbed. This included the charging ratio and power-consistency features. This procedure ensures that the synthetic attack modifies the underlying charging behavior while maintaining internally consistent relationships among the available operational variables. The synthetic attacks should therefore be interpreted as controlled emulations of documented EV charging attack mechanisms rather than as real cyberattack traces.

**Physical consistency constraints.** To avoid generating synthetic observations that are anomalous solely because of algebraic inconsistencies, the manipulated charging variables were generated under the physical relationship between energy, duration, and average power. Specifically, charging energy, duration, and average power were treated as coupled quantities according to E≈PD, and average power was recalculated after perturbing energy and duration. Energy and duration were constrained to positive values, while the resulting power values were checked against the operational range observed in the source charging dataset. Temporal features were derived from the corresponding charging timestamps rather than independently assigning incompatible temporal values. SOC was not available in the source dataset and therefore was not directly modeled. Derived cybersecurity-aware features were recalculated after manipulation so that they reflected the perturbed operational variables consistently. These constraints reduce the possibility that the detectors identify attacks solely through artificial algebraic inconsistencies introduced by the synthetic attack generator.

The cyberattack scenarios used in this study are synthetically generated because the underlying real-world EV charging dataset contains operational charging records but does not include verified cyberattack labels or documented attack incidents. To improve the realism of the synthetic scenarios, attack mechanisms were designed to represent documented EV charging threats. In particular, the stealth attack is conceptually aligned with charge manipulation attacks (CMAs), in which an adversary manipulates charging-related characteristics while attempting to preserve operational plausibility and avoid straightforward anomaly detection. The CMA threat model reported by Jahangir et al. [28] provides a literature-grounded basis for evaluating such charging manipulation behavior. Accordingly, the synthetic attacks in this study should be interpreted as controlled emulations of documented attack mechanisms rather than observations of actual cyberattack events.

### 3.6. Isolation Forest-Based Anomaly Detection

Isolation Forest was adopted as a baseline unsupervised anomaly detector to identify abnormal charging sessions under aggressive and stealth cyberattacks. The anomaly score is computed as follows:

(11) $$s(x,n)=2^{-\frac{E(h(x))}{c(n)}}\;$$

where h(x) denotes path length, c(n) is the normalization factor, and n is the number of observations. The model employed a contamination rate of 0.15, 100 estimators, and random_state = 42 for reproducibility and computational efficiency.

### 3.7. LSTM Autoencoder-Based Anomaly Detection

An LSTM autoencoder was developed to model sequential charging behavior and identify anomalies through reconstruction error. The encoder–decoder architecture learned latent charging representations, while anomalous sessions produced larger reconstruction errors. Reconstruction loss was computed using the Mean Squared Error (MSE):

(12) $$L=\frac{1}{N}\sum_{i=1}^{N}(x_{i}-{\hat{x}}_{i}{)}^{2}\;$$

where $x_{i}$ and ${\hat{x}}_{i}$ denote original and reconstructed charging features, respectively, and N is the number of samples. The model employed 32 hidden units, a batch size of 32, Adam optimization, and 20 training epochs. Reconstruction thresholds were applied to classify anomalous charging sessions.

### 3.8. Federated Learning Framework

A privacy-preserving federated learning framework enabled distributed cybersecurity intelligence without centralized charging data collection. The federated environment consisted of four charging clients (K=4) representing heterogeneous EV charging stations under non-IID conditions. Each client independently trained a local LSTM autoencoder using localized data and exchanged only model parameters. Global aggregation followed Federated Averaging (FedAvg):

(13) $$\begin{array}{cccc} & w_{t+1}=\sum_{k=1}^{K}\frac{n_{k}}{n}w_{t}^{k} & & \end{array}$$

where $w_{t}^{k}$ denotes local model parameters, $n_{k}$ is local dataset size, K is the number of clients, and n is the total sample size. Federated training employed 4 communication rounds, 32 hidden units, 32 batches, 20 epochs, MSE loss, and Adam optimization.

A data-localized federated learning framework was implemented to enable distributed cybersecurity intelligence without directly centralizing raw charging records. The federated environment consisted of four charging clients representing heterogeneous EV charging environments under non-IID conditions. Each client independently trained a local LSTM autoencoder using its localized charging data, while only model parameters were exchanged with the central server. Global aggregation was performed using Federated Averaging (FedAvg). Federated training consisted of four communication rounds, with all four clients participating in every round. At the beginning of the federated process, a common LSTM autoencoder was initialized at the central server and distributed to all participating clients. During each communication round, each client trained the received global model for 20 local epochs using its local training data. The resulting model parameters were then transmitted to the central server for FedAvg aggregation, and the updated global model was redistributed for the subsequent round. Thus, each client performed 20 local epochs per communication round, corresponding to 80 local epochs across the four communication rounds. The final global model obtained after the fourth aggregation round was used for federated evaluation.

Convergence was monitored through the local training loss trajectories across communication rounds. Differences in loss reduction among clients were expected because each client operated on a distinct non-IID local charging distribution. The final federated model was selected after the fourth global aggregation without additional centralized fine-tuning. The four-round configuration was selected to provide repeated local optimization and global aggregation while maintaining a computationally tractable experimental setting.

The privacy-related scope of the framework is limited to data locality and the absence of direct raw-record sharing. Although raw charging sessions were not transmitted during federated training, secure aggregation, differential privacy, and formal leakage-resistance mechanisms were not implemented. Potential inference risks from exchanged model parameters, including gradient or parameter leakage and membership inference, were not experimentally evaluated, and malicious server or malicious client attacks were outside the scope of the present threat model. Accordingly, the framework is not intended to provide a formal privacy guarantee against an honest-but-curious or malicious server or participating clients. These limitations should be considered when interpreting the privacy-related contribution of the proposed approach.

The four-client configuration was designed as a proof-of-concept evaluation of decentralized anomaly detection under heterogeneous charging conditions rather than as a comprehensive scalability study. The present evaluation does not quantitatively characterize non-IID severity using formal distribution distance measures or provide a complete client-level analysis of sample counts, attack prevalence, and detection performance. Communication traffic in bytes and end-to-end local training time were also not recorded. Therefore, the four-client experiment demonstrates the feasibility of federated anomaly detection under heterogeneous local data but does not establish scalability to large numbers of charging stations or clients. Quantitative heterogeneity measures, client-level performance analysis, communication-cost profiling, training-time measurements, and experiments involving progressively larger client populations are required for comprehensive scalability validation in future work.

### 3.9. Performance Evaluation Metrics

Performance was evaluated using accuracy, precision, recall, F1-score, and ROC-AUC under aggressive and stealth cyberattack scenarios.

(14) $$Accuracy=\frac{TP+TN}{TP+TN+FP+FN}\;$$

(15) $$Precision=\frac{TP}{TP+FP}\;$$

(16) $$Recall=\frac{TP}{TP+FN}\;$$

(17) $$F1=\frac{2\times Precision\times Recall}{Precision+Recall}\;$$

where TP, TN, FP, and FN denote true positive, true negative, false positive, and false negative predictions, respectively. ROC-AUC, confusion matrices, and ROC curves were additionally used to evaluate anomaly separability, false alarms, and model robustness.

Performance was evaluated using accuracy, precision, recall, F1-score, and ROC-AUC under aggressive and stealth cyberattack scenarios, where TP, TN, FP, and FN denote true positive, true negative, false positive, and false negative predictions, respectively. Accuracy, precision, recall, and F1-score were calculated from the confusion matrix to quantify classification performance, while ROC-AUC and ROC curves were used to assess anomaly discrimination across classification thresholds. Confusion matrices were additionally examined to characterize false positive and false negative behavior. Because the present study uses session-level synthetic attack scenarios and does not model continuous real-time attack streams, operational metrics such as detection delay and false alarms per charger-day were not directly evaluated. Similarly, the current experimental protocol did not include repeated-seed confidence intervals or systematic variation in federated client partitions. These aspects are therefore considered limitations of the current evaluation and are identified as priorities for subsequent validation using larger and temporally continuous charging datasets.

### 3.10. Experimental Configuration

Experiments were implemented in Python 3.10 using Google Colab, with Pandas 2.2.2, NumPy 1.26.4, Matplotlib 3.9.2, Seaborn 0.13.2, Scikit-learn 1.5.2, TensorFlow 2.17.1, and Keras 3.5.0 for data processing, visualization, machine learning, and deep learning model development. The dataset was split using an 80:20 stratified train–test ratio. Isolation Forest employed a contamination ratio of 0.15 and random seed 42, while the LSTM autoencoder used 32 hidden units, a batch size of 32, Adam optimization, and 20 epochs. Federated learning was conducted using 4 distributed clients and 4 communication rounds under non-IID charging behavior.

To distinguish the evaluation protocols used for centralized and federated learning, the centralized stealth attack experiments were evaluated using the full augmented evaluation set containing 1795 samples, comprising 1526 normal sessions and 269 synthetic stealth attack samples. In contrast, the federated LSTM was evaluated using a fixed smaller evaluation subset of 97 samples, consisting of 70 normal and 27 stealth attack samples. This difference reflects the distinct experimental evaluation protocols: the centralized model was assessed using the larger augmented dataset, whereas the federated model was evaluated on a fixed subset to provide a consistent test set for the aggregated global model under decentralized training. The 97-sample federated evaluation set should therefore not be interpreted as the direct 20% test partition of the original 1929-session dataset.

The attack configuration was kept independent of the model hyperparameter configuration to avoid coupling the attack generation process with the detector optimization. The approximately 14.0% attack injection proportion was applied consistently across the aggressive and stealth scenarios, while the attack severity was controlled through the perturbation mechanism. The detection models were then trained and evaluated using the same architectural and optimization settings across the corresponding attack conditions. Isolation Forest used a contamination parameter of 0.15 as an unsupervised baseline, whereas the LSTM autoencoder used 32 hidden units, a batch size of 32, Adam optimization, and 20 training epochs. The federated LSTM used the same local LSTM configuration across four clients and four communication rounds, with only model parameters exchanged through FedAvg. Thus, the attack injection proportion and perturbation intensity define the cybersecurity evaluation condition, whereas the model configuration defines the learning and detection capacity, allowing the effect of attack characteristics on detection performance to be evaluated without changing the detector architecture or training protocol.

The centralized and federated LSTM models did not receive identical nominal optimization schedules. The centralized model was trained for 20 epochs on the centralized training data, whereas each federated client performed 20 local epochs during each of four communication rounds, resulting in 80 local epochs per client over the complete federated training process. However, the federated optimization was distributed across four clients and interrupted by global parameter aggregation after each communication round, whereas the centralized model optimized directly over the complete centralized training distribution. Therefore, the epoch counts should not be interpreted as equivalent measures of computational or optimization effort. The comparison is intended to evaluate the practical difference between centralized and decentralized training under their respective learning protocols rather than to claim strictly matched optimization budgets.

## 4. Results

### 4.1. Exploratory Analysis of Ev Charging Behavior

**Figure 2:**
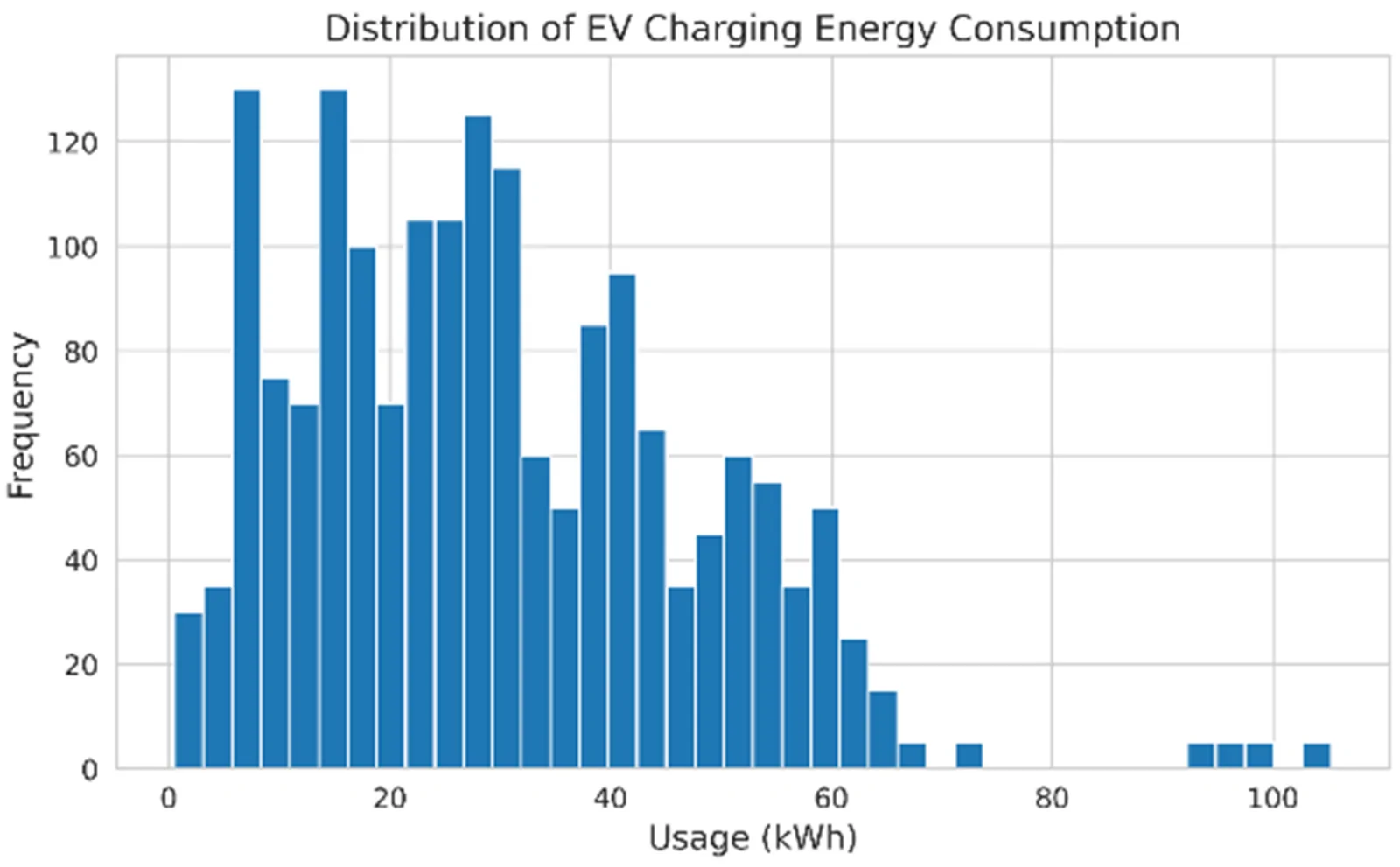
Distribution of charging energy consumption in the charging dataset.

Prior to cybersecurity experimentation, exploratory statistical analysis was conducted to examine EV charging behavior, including charging energy consumption, duration, charging power, temporal activity, and feature relationships, snd characterize legitimate charging patterns before anomaly detection modeling:

As shown in Figure 2, charging energy exhibited a right-skewed distribution, with most sessions concentrated at moderate energy levels and fewer high-demand charging events. This reflects realistic EV charging behavior and highlights operational variability relevant to cybersecurity analysis, as anomalous sessions may manipulate energy consumption patterns to mimic legitimate charging behavior.

**Figure 3:**
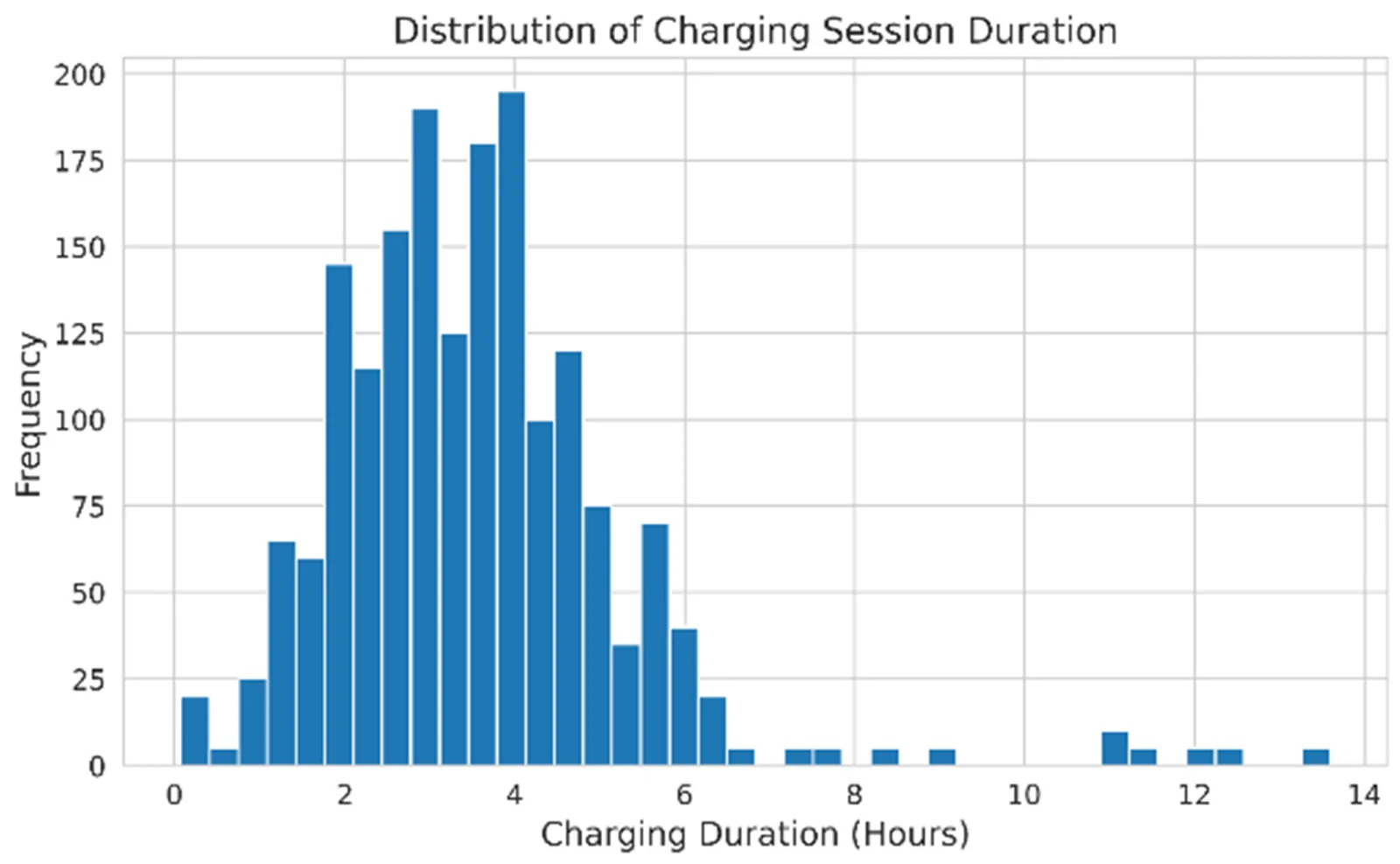
Distribution of EV charging session duration.

As shown in Figure 3, most charging sessions were concentrated within short-to-medium durations, while fewer long-duration sessions formed a long-tail distribution. This temporal variability reflects diverse EV charging behavior and provides useful context for anomaly detection, as cyberattacks may manipulate charging duration to alter charging profiles or evade detection.

**Figure 4:**
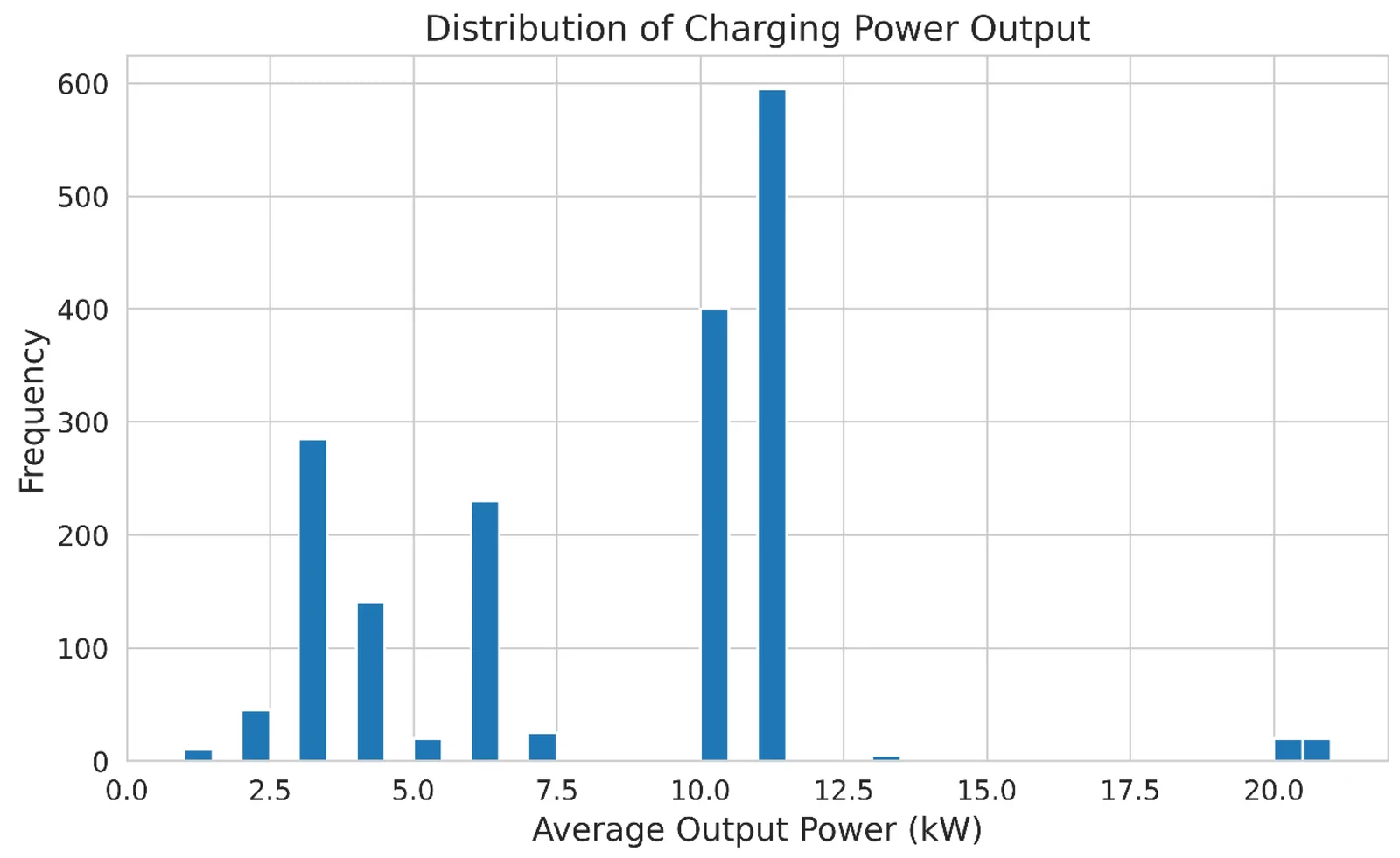
Distribution of charging power output in EV charging infrastructure.

As shown in Figure 4, the charging power distribution exhibited multiple discrete clusters, indicating heterogeneous charging states and varying power delivery conditions. This variability highlights the need for adaptive anomaly detection mechanisms capable of distinguishing legitimate charging behavior from malicious manipulation.

**Figure 5:**
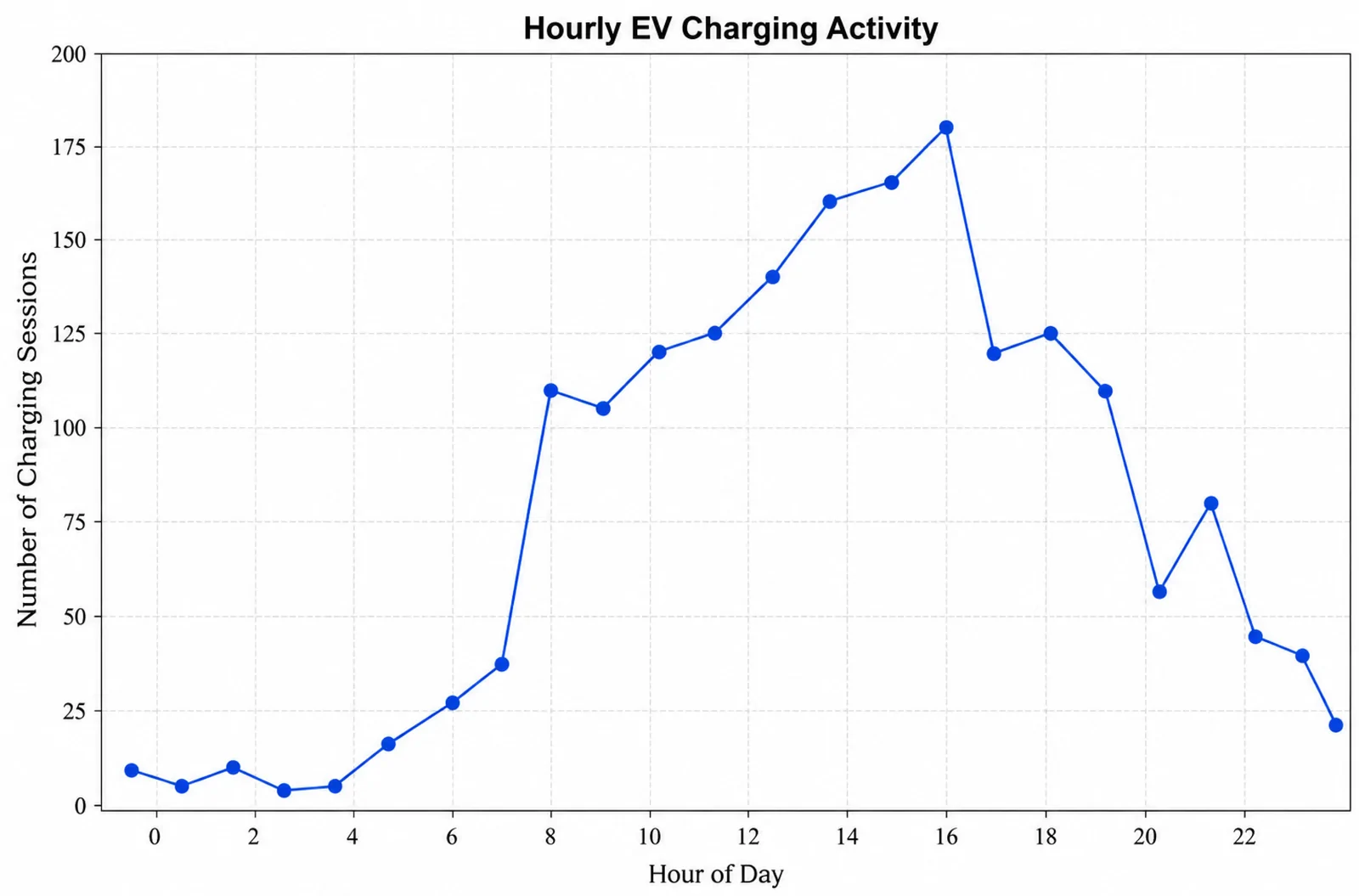
Hourly EV charging activity across the charging infrastructure.

As illustrated in Figure 5, charging activity varied throughout the day, increasing during daytime and decreasing at night, reflecting realistic charging behavior patterns. These temporal characteristics are important for cybersecurity analysis, as abnormal activity outside expected operating periods may indicate malicious manipulation or unauthorized charging access.

**Figure 6:**
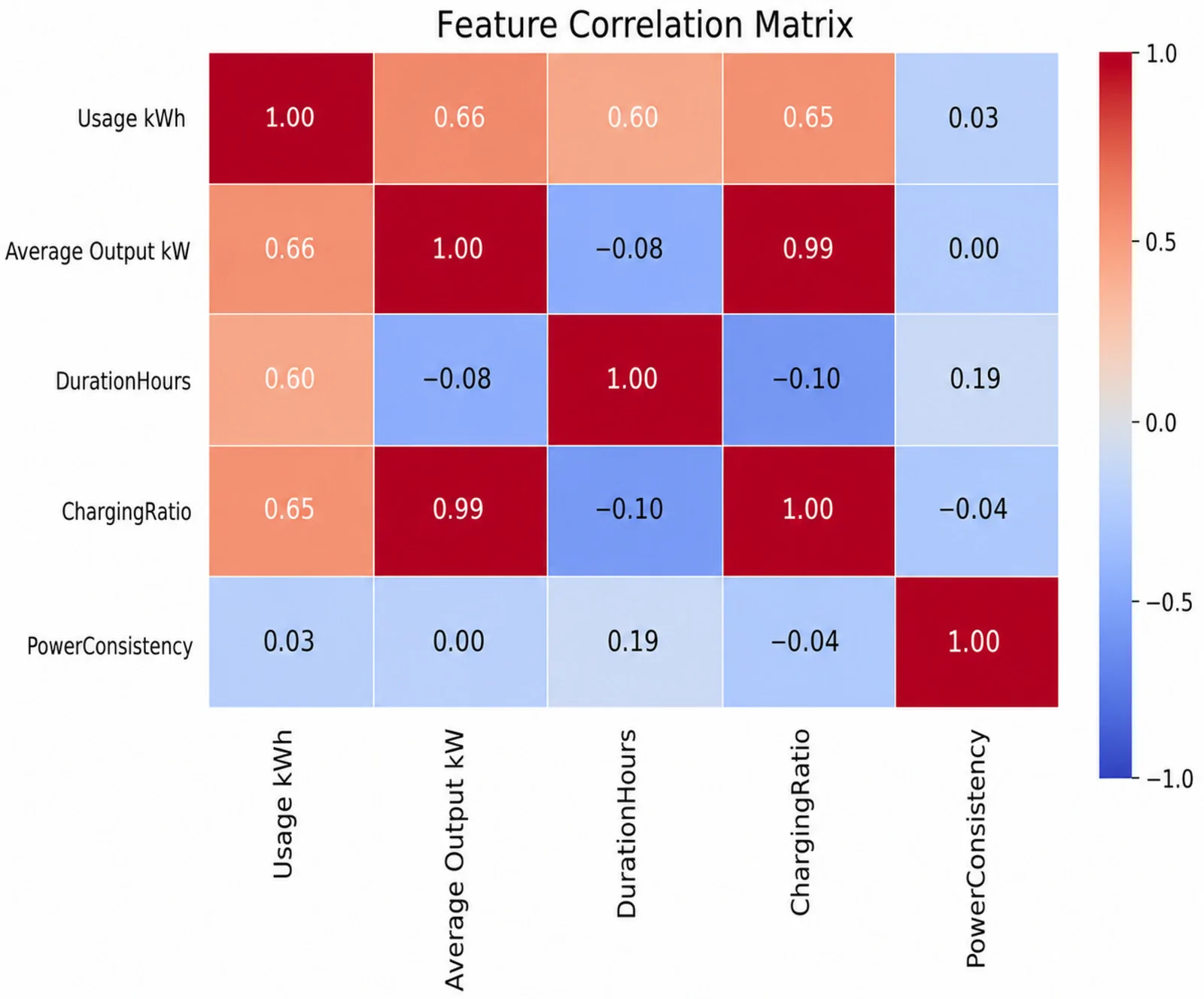
Correlation matrix of cybersecurity-aware EV charging features.

As shown in Figure 6, feature correlation analysis revealed meaningful relationships among charging variables, including strong correlations between charging ratio and average charging power and moderate correlations between charging energy consumption and charging duration. These engineered cybersecurity-aware features improved behavioral separability for anomaly detection and cyberattack characterization.

Table 2 summarizes the descriptive statistics of EV charging features. Charging energy consumption showed substantial variability, with a mean of 35.55 kWh and a maximum of 229.69 kWh, while charging duration averaged 3.24 h. Charging power exhibited heterogeneous behavior across sessions. Engineered features, including charging ratio and power consistency, also showed notable variability, supporting their suitability for anomaly detection and cyberattack characterization.

### 4.2. Aggressive Cyberattack Detection Performance

To evaluate anomaly detection under highly disruptive adversarial conditions, an aggressive cyberattack scenario was introduced through substantial manipulation of charging behavior features, including energy consumption, charging duration, power, and temporal activity. These attacks emulate false data injections and malicious charging manipulation, introducing strong operational inconsistencies and highly separable attack conditions.

**Figure 7:**
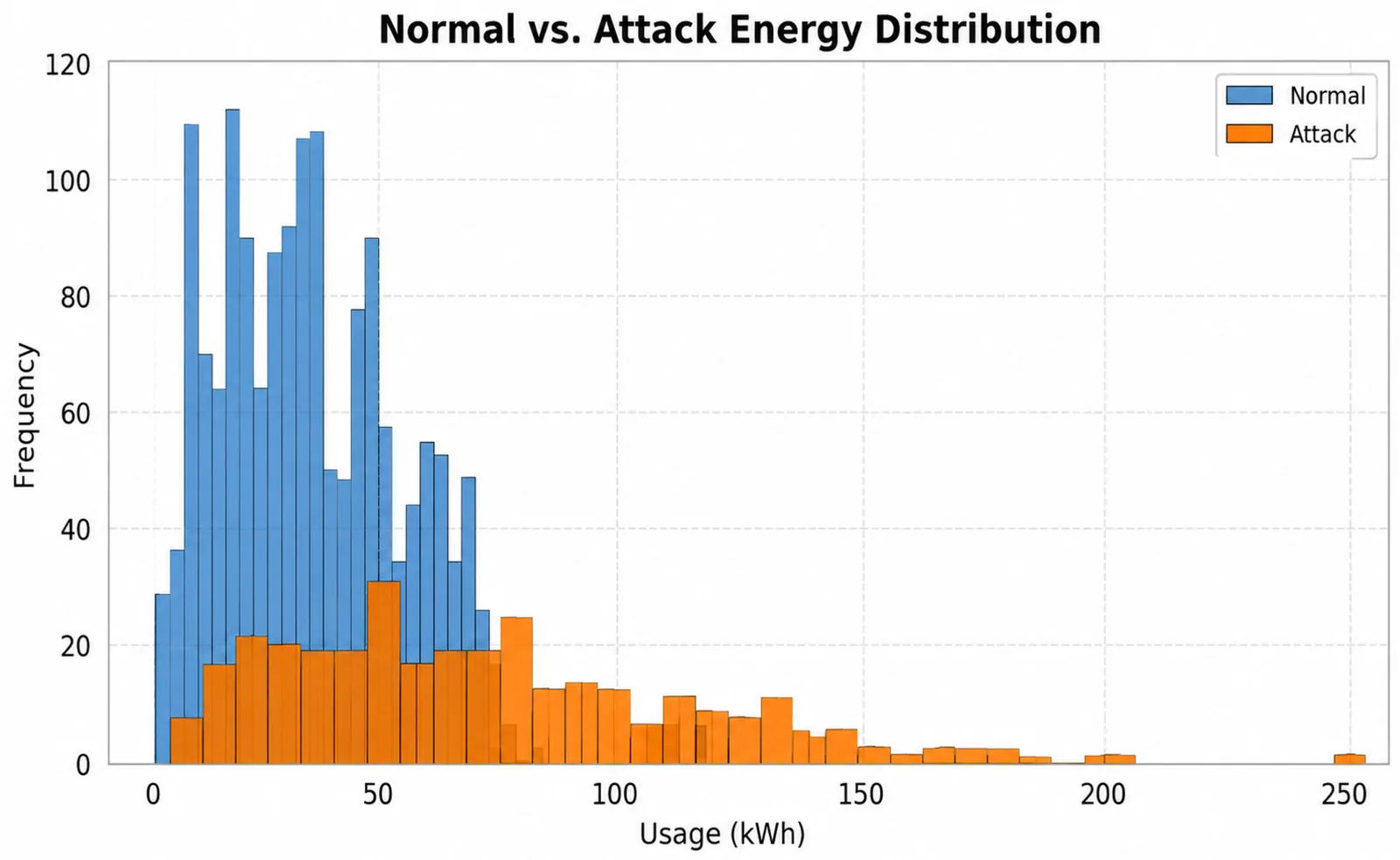
Comparison between normal charging behavior and aggressive cyberattack manipulation.

As illustrated in Figure 7, aggressive cyberattacks introduced substantial deviations from legitimate charging behavior, resulting in clear separation between normal and malicious charging sessions. The pronounced behavioral inconsistency made aggressive attacks comparatively easier to detect.

Table 3 summarizes the aggressive cyberattack configuration used for experimental evaluation. A total of 269 charging sessions were synthetically manipulated, corresponding to an attack injection proportion of approximately 14.0% relative to the 1929-session dataset. The same number of attack samples was used for the aggressive and stealth scenarios to maintain a consistent attack sample size across the experimental conditions. The aggressive configuration introduced substantial perturbations to charging behavior to represent highly disruptive adversarial conditions.

### 4.3. Isolation Forest-Based Detection Under Aggressive Attacks

**Figure 8:**
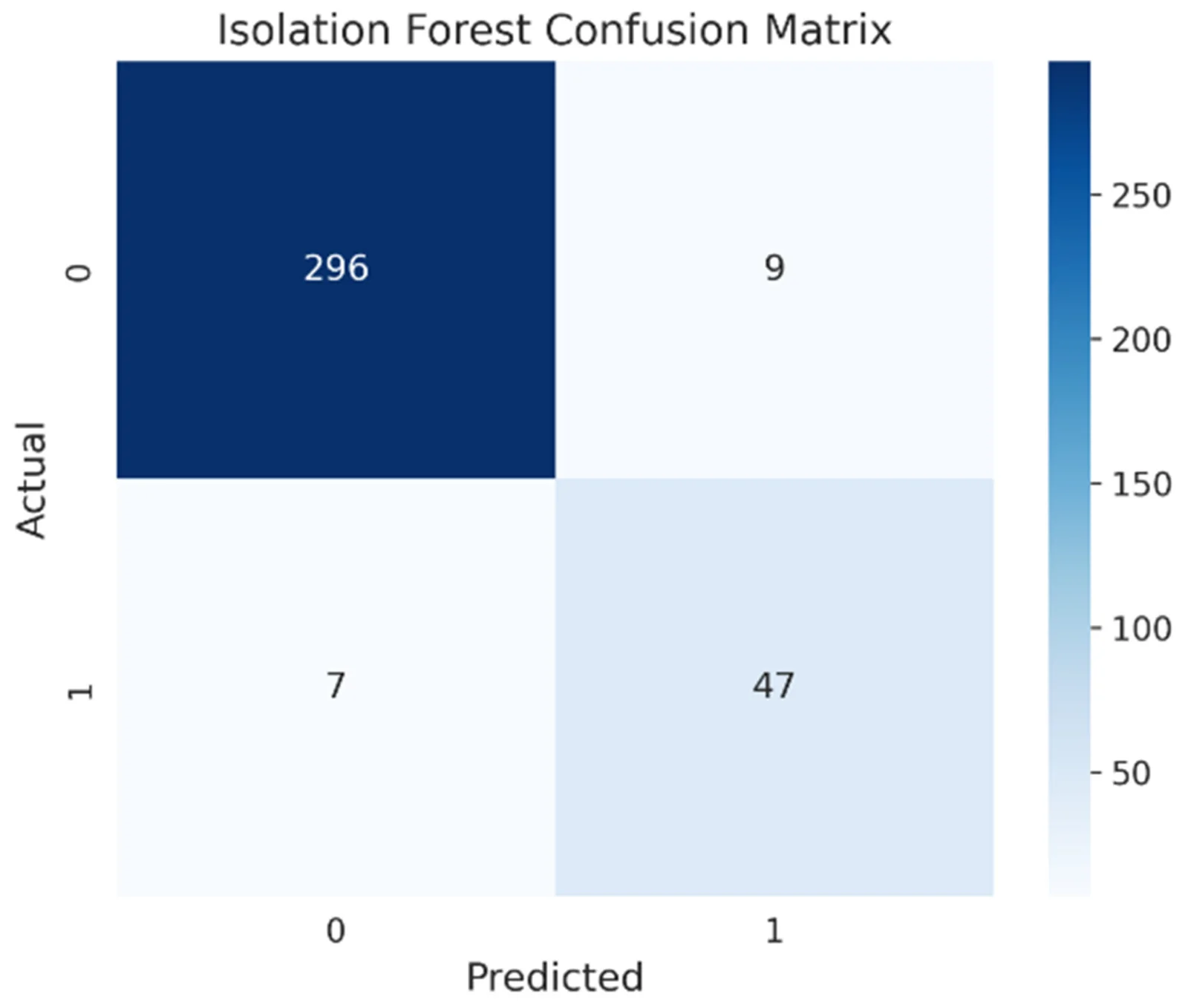
Confusion matrix of Isolation Forest under aggressive cyberattack conditions.

**Figure 9:**
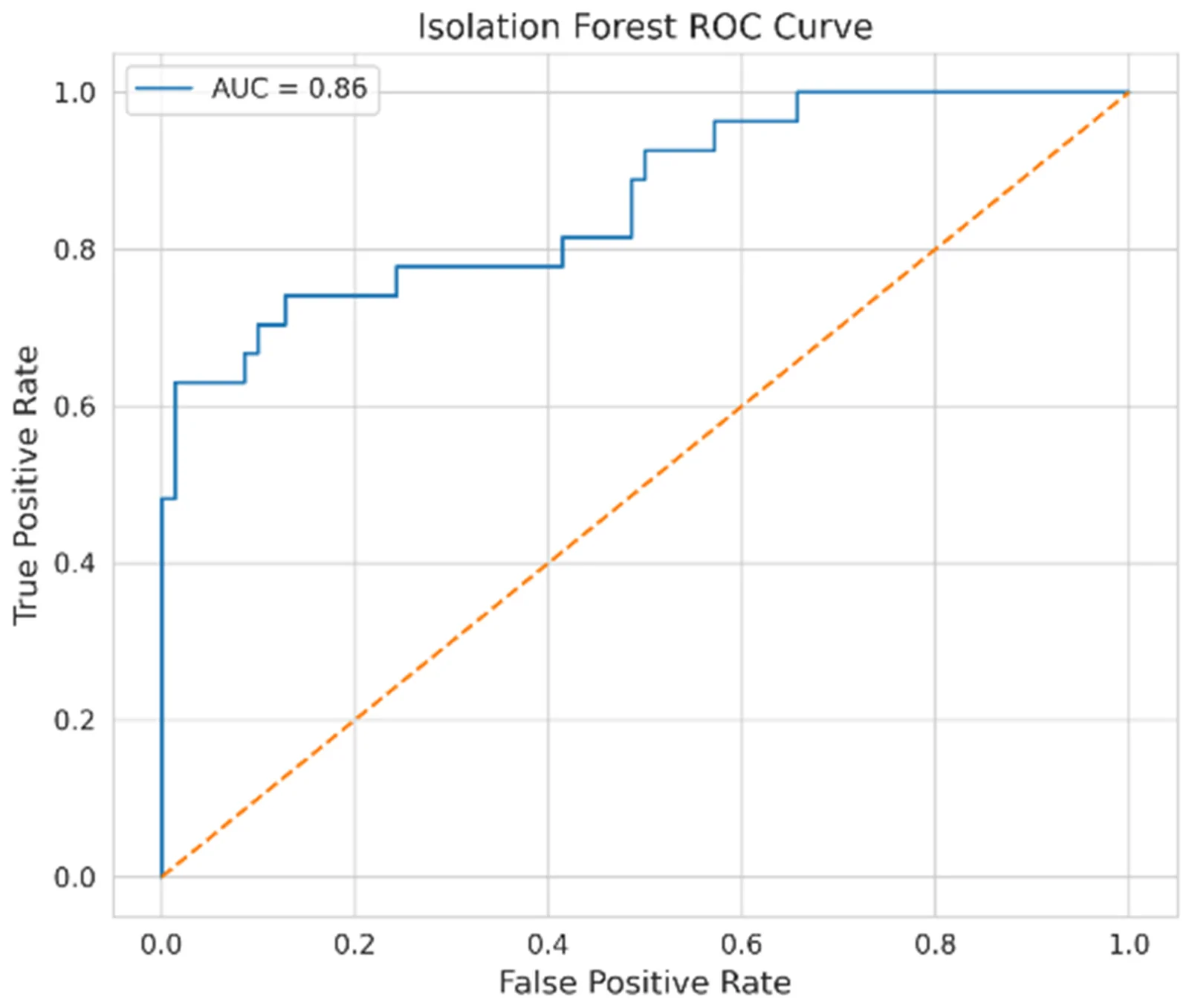
Receiver operating characteristic (ROC) curve of Isolation Forest anomaly detection under aggressive cyberattack conditions.

As shown in Figure 8, Isolation Forest demonstrated strong anomaly detection capability under aggressive cyberattack conditions. The confusion matrix revealed high classification performance with limited false positive and false negative predictions, indicating effective behavioral separation between malicious and legitimate charging sessions. Furthermore, the receiver operating characteristic (ROC) curve shown in Figure 9 demonstrated strong classification capability with an area under the (AUC) value of 0.86, indicating robust anomaly separability under highly manipulated charging conditions.

The experimental findings summarized in Table 4 indicate that Isolation Forest achieved strong detection performance with 96% classification accuracy and an attack F1-score of 0.85. The high recall value demonstrates strong capability for identifying malicious charging behavior, while limited false alarm behavior suggests effective anomaly separation under aggressive attack conditions. These findings indicate that classical anomaly detection techniques remain highly effective when malicious charging behavior introduces substantial operational inconsistencies.

### 4.4. Centralized LSTM Autoencoder Under Aggressive Attacks

**Figure 10:**
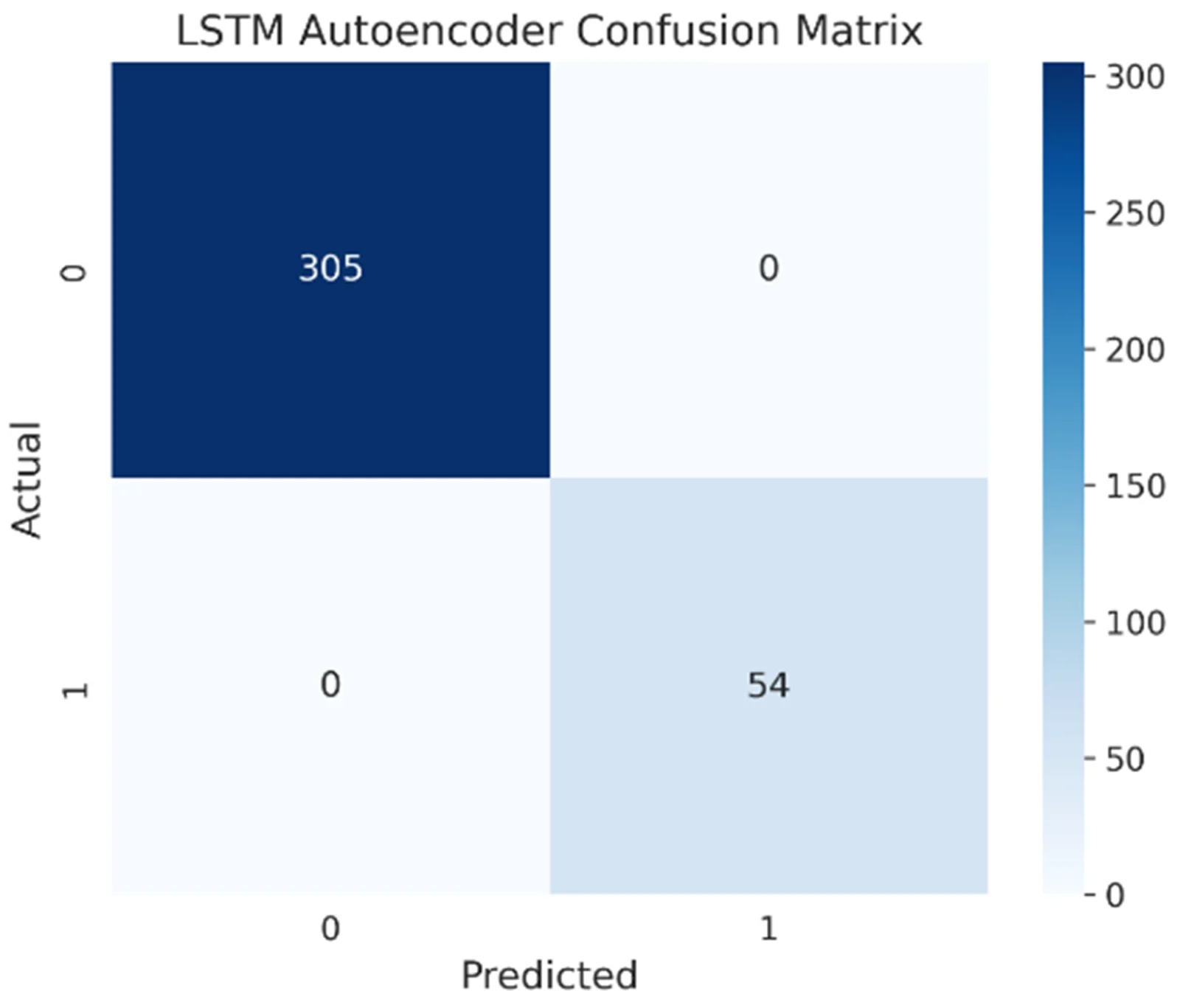
Confusion matrix of centralized LSTM autoencoder anomaly detection under aggressive cyberattack conditions.

As shown in Figure 10, the centralized LSTM autoencoder achieved near-perfect anomaly detection capability under aggressive cyberattack conditions. The reconstruction-based deep learning framework successfully learned normal charging behavior and accurately detected manipulated charging sessions through reconstruction error analysis.

Table 5 shows that the centralized LSTM autoencoder achieved 100% classification accuracy and perfect attack detection performance under aggressive attack conditions. While these results demonstrate the effectiveness of deep anomaly detection architectures under strongly separable attack distributions, such performance primarily reflects idealized experimental conditions rather than realistic adversarial environments in which malicious charging behavior may intentionally remain statistically similar to legitimate charging sessions.

### 4.5. Stealth Cyberattack Detection Performance

To create a more realistic cybersecurity evaluation environment, stealth cyberattacks were generated through subtle perturbation of charging behavior characteristics. Unlike aggressive attacks, stealth cyberattacks introduced only minor modifications to charging parameters while remaining statistically similar to legitimate charging sessions. Such attacks emulate intelligent adversarial behavior in which malicious charging manipulation is intentionally designed to evade anomaly detection mechanisms while preserving operational plausibility.

**Figure 11:**
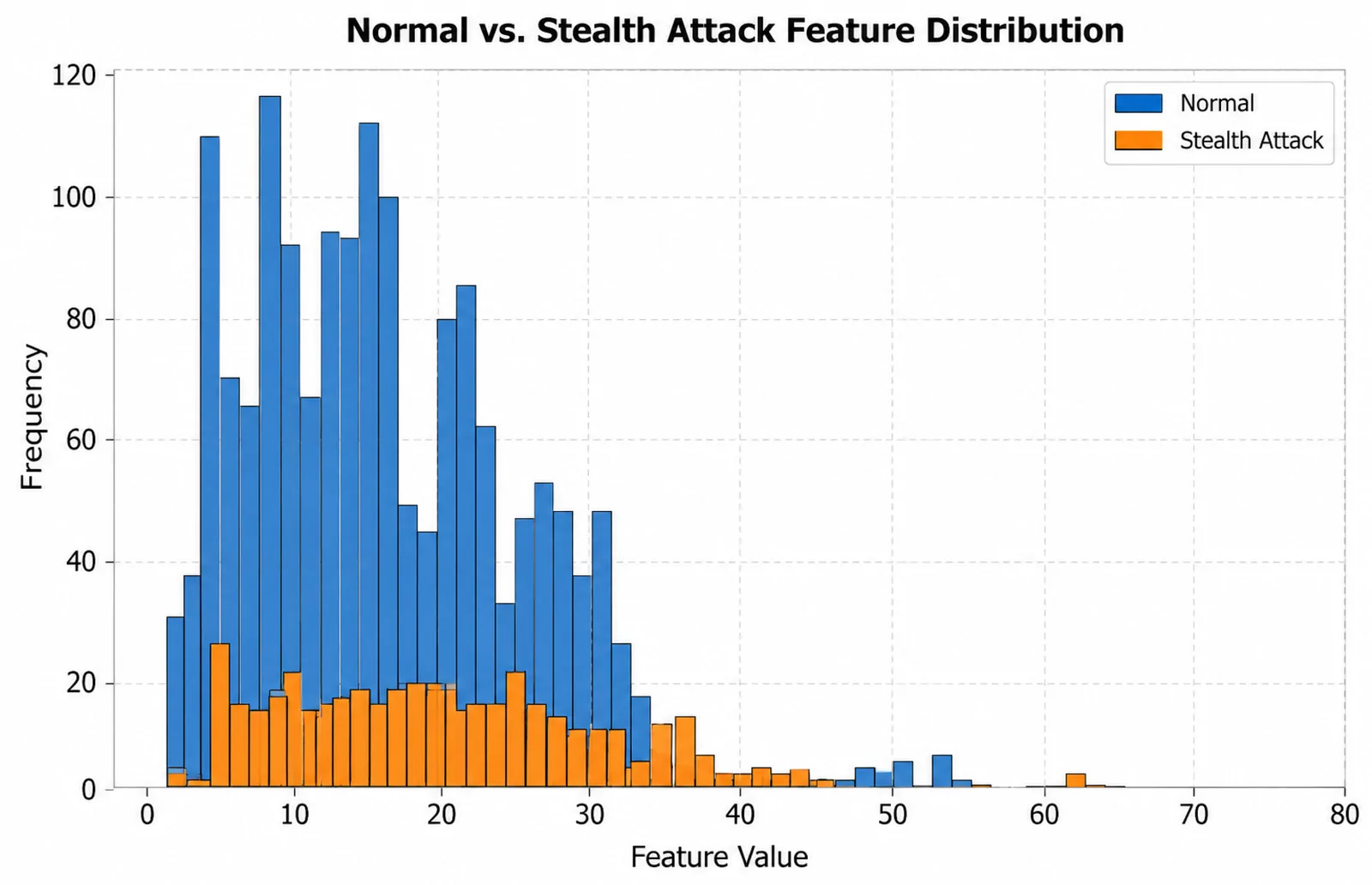
Comparison between normal charging behavior and stealth cyberattack manipulation.

As illustrated in Figure 11, stealth cyberattacks exhibited substantial statistical overlap with legitimate charging behavior, thereby significantly increasing anomaly detection complexity. Unlike aggressive cyberattacks that introduced strong operational inconsistencies, stealth attacks remained behaviorally similar to normal charging sessions, reducing anomaly separability and increasing classification difficulty. Such behavior more realistically reflects practical cyberattack scenarios in IoT-enabled EV charging environments, where malicious actors attempt to remain undetected while gradually manipulating charging operations.

Table 6 summarizes the stealth cyberattack configuration adopted in the experimental evaluation. A total of 269 charging sessions were synthetically manipulated, corresponding to the same approximately 14.0% attack injection proportion used for the aggressive scenario. Maintaining an identical number of attack samples across the two scenarios ensures that the primary difference between aggressive and stealth evaluations is the attack characteristic and perturbation intensity rather than the number of injected attack instances. For the stealth scenario, energy and duration were perturbed within a bounded ±10% range to preserve similarity with legitimate charging behavior and emulate difficult-to-detect charging manipulation.

### 4.6. Validation Against a Literature-Grounded Charge Manipulation Attack

Because the underlying EV charging dataset does not contain verified cyberattack events, an additional validation was conducted to assess whether the synthetic stealth attack design is consistent with a documented EV charging threat. The validation was based on the charge manipulation attack (CMA) scenario reported by Jahangir et al. [28], in which an attacker manipulates charging-related information to alter EV charging behavior while maintaining operational plausibility and reducing the likelihood of immediate detection. This threat model is particularly relevant to smart EV charging because charging behavior can be manipulated without necessarily producing the large statistical deviations associated with highly disruptive attacks.

The proposed stealth attack was therefore evaluated as a literature-grounded emulation of this CMA scenario. In the synthetic validation, charging behavior was subjected to low-intensity manipulation while preserving the general statistical characteristics of legitimate charging sessions. This design is consistent with the objective of CMAs, which is to modify charging behavior without producing conspicuous deviations from normal operational patterns. The existing stealth attack formulation uses bounded low-intensity perturbations specifically to maintain operational plausibility and increase the overlap between malicious and legitimate charging behavior.

The validation demonstrates that the proposed stealth attack scenario represents a plausible implementation of a documented EV charging attack mechanism rather than an arbitrary numerical perturbation. The resulting attack behavior retained substantial similarity to legitimate charging sessions, thereby producing the increased detection difficulty observed in the stealth experiments. In particular, the Isolation Forest attack F1-score decreased to 0.61 under the stealth scenario, whereas the centralized LSTM and federated LSTM achieved attack F1-scores of 0.96 and 0.67, respectively. These results are consistent with the expected characteristics of stealthy charge manipulation, where subtle changes are more difficult to isolate using conventional statistical anomaly detection methods.

This validation should not be interpreted as validation using real cyberattack traces. Rather, it establishes that the synthetic stealth scenario is grounded in a documented EV charging attack mechanism and exhibits the expected behavioral characteristics of charge manipulation. The absence of labeled real-world cyberattack records remains a limitation of the present study and prevents direct assessment of the proposed framework against operational attack logs.

### 4.7. Isolation Forest-Based Detection Under Stealth Attacks

**Figure 12:**
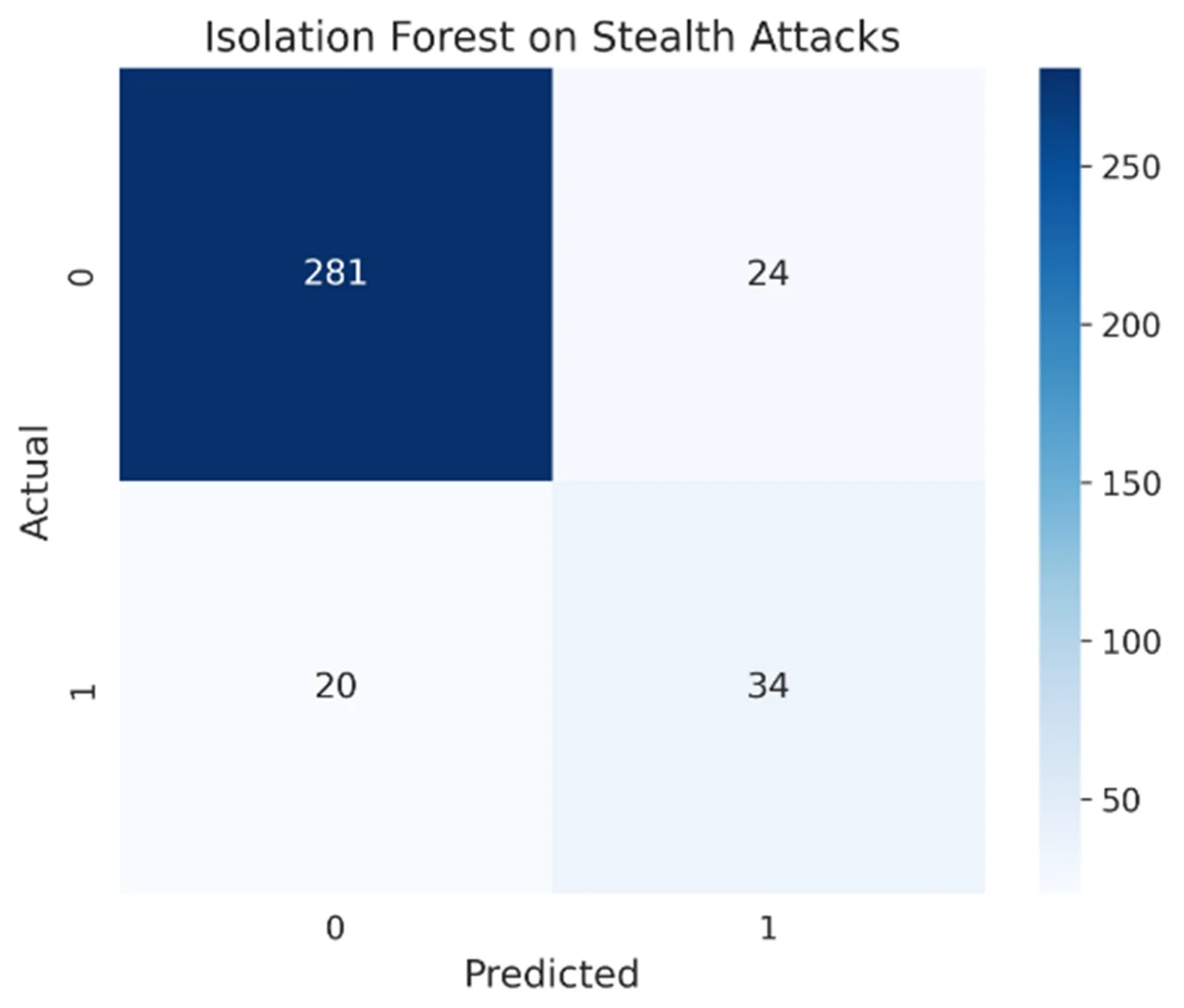
Confusion matrix of Isolation Forest under stealth cyberattacks conditions.

As shown in Figure 12, Isolation Forest exhibited noticeable performance degradation under stealth cyberattack conditions relative to aggressive attack scenarios. The confusion matrix revealed increased false negative and false positive predictions due to stronger statistical similarity between malicious and legitimate charging behavior.

The experimental results summarized in Table 7 demonstrate that Isolation Forest performance degraded under stealth cyberattack conditions, achieving 88% classification accuracy, 0.59 precision, 0.63 recall, and an attack F1-score of 0.61. Compared with aggressive cyberattacks, the reduction in precision, recall, and F1-score demonstrates that classical anomaly detection approaches face substantial difficulty identifying intelligent adversarial charging manipulation designed to imitate legitimate operational charging behavior.

### 4.8. Centralized LSTM Autoencoder Under Stealth Attacks

To evaluate deep learning-based anomaly detection capability under realistic adversarial conditions, the centralized LSTM autoencoder was further assessed using stealth cyberattack scenarios characterized by subtle manipulation of EV charging behavior. Unlike aggressive attacks that generate substantial operational deviations, stealth attacks remained statistically similar to legitimate charging sessions, thereby increasing anomaly detection complexity.

**Figure 13:**
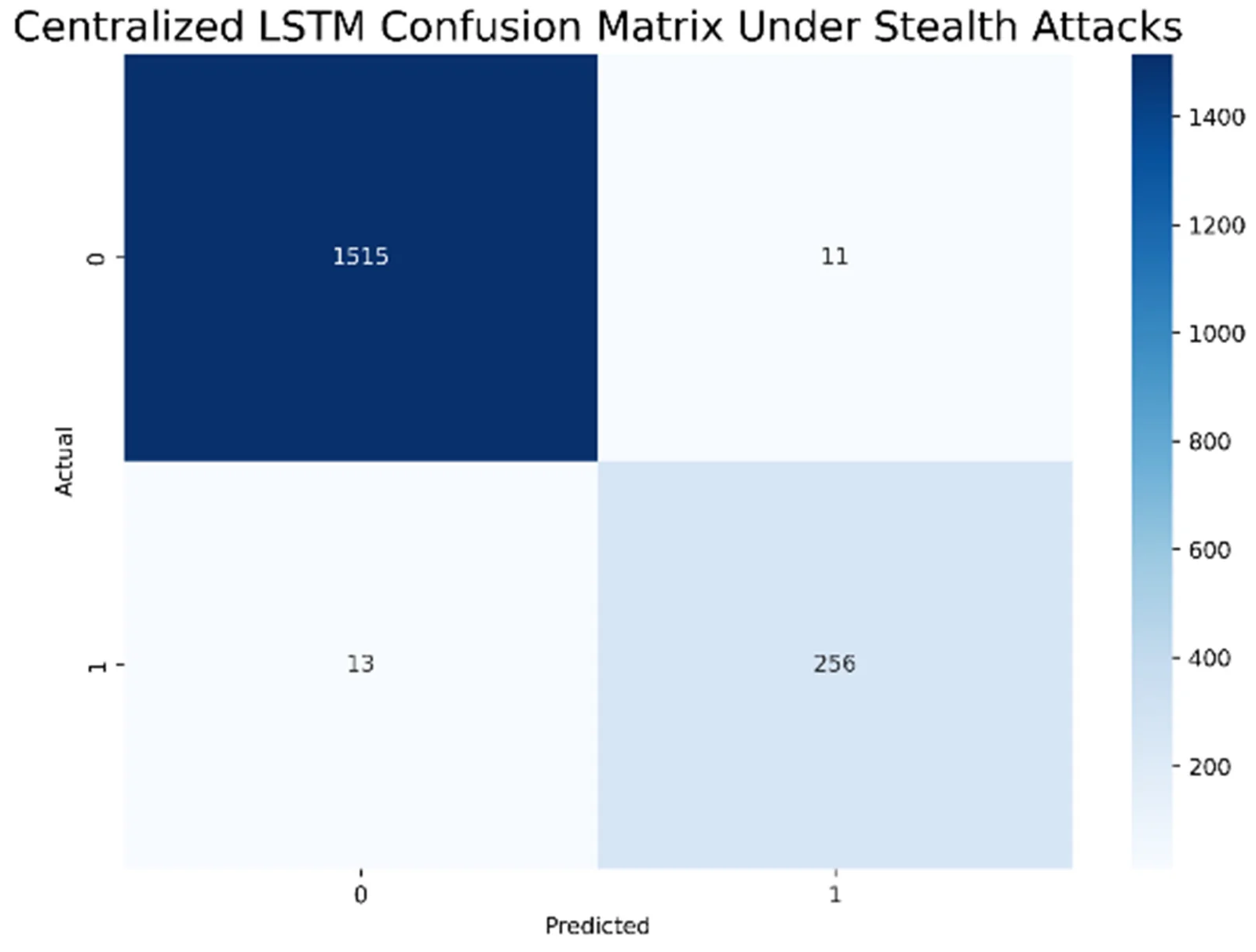
Confusion matrix of centralized LSTM autoencoder anomaly detection under stealth cyberattacks conditions.

The centralized LSTM autoencoder demonstrated strong resilience against stealth cyberattacks by learning latent temporal charging representations and identifying subtle deviations through reconstruction error analysis. As illustrated in Figure 13, the proposed LSTM-based anomaly detection framework successfully distinguished malicious charging sessions while maintaining low false alarm behavior. The confusion matrix revealed 1515 correctly identified normal charging sessions and 256 correctly detected stealth cyberattacks, with only limited false positives and false negatives.

Figure 13 further demonstrates that reconstruction-based deep learning models exhibit strong behavioral sensitivity to subtle charging inconsistencies that may remain difficult to capture using statistical anomaly detection approaches.

The quantitative evaluation results shown in Table 8 indicate that the centralized LSTM autoencoder achieved an attack precision of 0.96, recall of 0.95, and F1-score of 0.96, with an overall classification accuracy of 0.99 under stealth cyberattack conditions. Compared with Isolation Forest-based detection, the centralized LSTM framework exhibited substantially stronger robustness against intelligent charging manipulation designed to mimic normal EV charging behavior.

These findings suggest that deep sequence learning models offer improved cybersecurity resilience for EV charging infrastructures operating under realistic cyberattack environments where malicious charging activity attempts to remain operationally inconspicuous.

### 4.9. Federated Cybersecurity Performance

A federated learning-based anomaly detection framework was implemented to preserve charging data privacy while enabling distributed cybersecurity intelligence across IoT-enabled EV charging nodes. Unlike centralized learning, federated learning enables distributed clients to independently train local anomaly detection models while sharing only model parameters with a central server.

In the proposed framework, local edge clients trained LSTM-based anomaly detection models using localized charging behavior. Instead of transmitting raw charging session data, only updated model weights were exchanged through the Federated Averaging (FedAvg) mechanism, reducing centralized data exposure while preserving privacy and supporting distributed cybersecurity intelligence.

**Figure 14:**
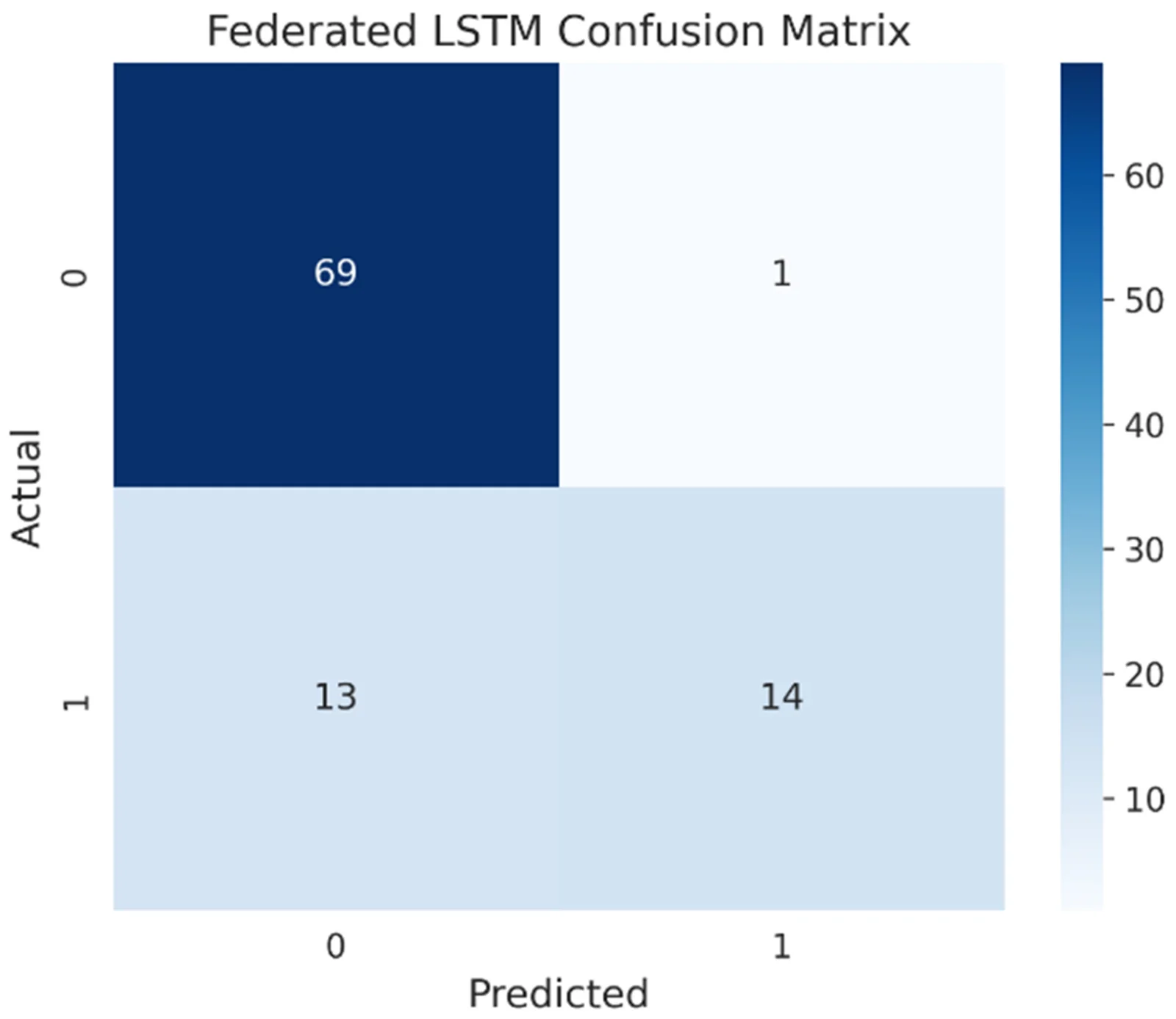
Confusion matrix of federated LSTM anomaly detection under stealth cyberattacks conditions.

The federated LSTM framework was evaluated under stealth cyberattack conditions to investigate detection robustness within a realistic distributed EV charging environment. As illustrated in Figure 14, the federated anomaly detection framework successfully detected stealth cyberattacks while maintaining reasonable classification performance despite operating under decentralized learning constraints. The confusion matrix demonstrates that the federated model correctly classified 69 normal charging sessions and 14 stealth attack sessions, while exhibiting moderate false positive and false negative behavior.

Unlike the centralized evaluation, which used the larger augmented stealth dataset, the federated LSTM was evaluated on a fixed 97-sample subset comprising 70 normal and 27 attack sessions. The smaller evaluation set was used consistently for the federated global-model assessment to maintain a controlled evaluation condition following decentralized training. Consequently, the support values reported in Table 9 are not directly comparable in magnitude with those reported for the centralized model; comparisons should instead focus on the corresponding performance metrics under their respective evaluation protocols.

The quantitative performance results presented in Table 9 indicate that the federated LSTM model achieved an attack precision of 0.93, recall of 0.52, and F1-score of 0.67, with an overall classification accuracy of 0.86. Although this performance remained lower than the centralized LSTM autoencoder under identical stealth attack conditions, the federated approach still demonstrated meaningful cybersecurity capability while preserving decentralized data privacy.

**Figure 15:**
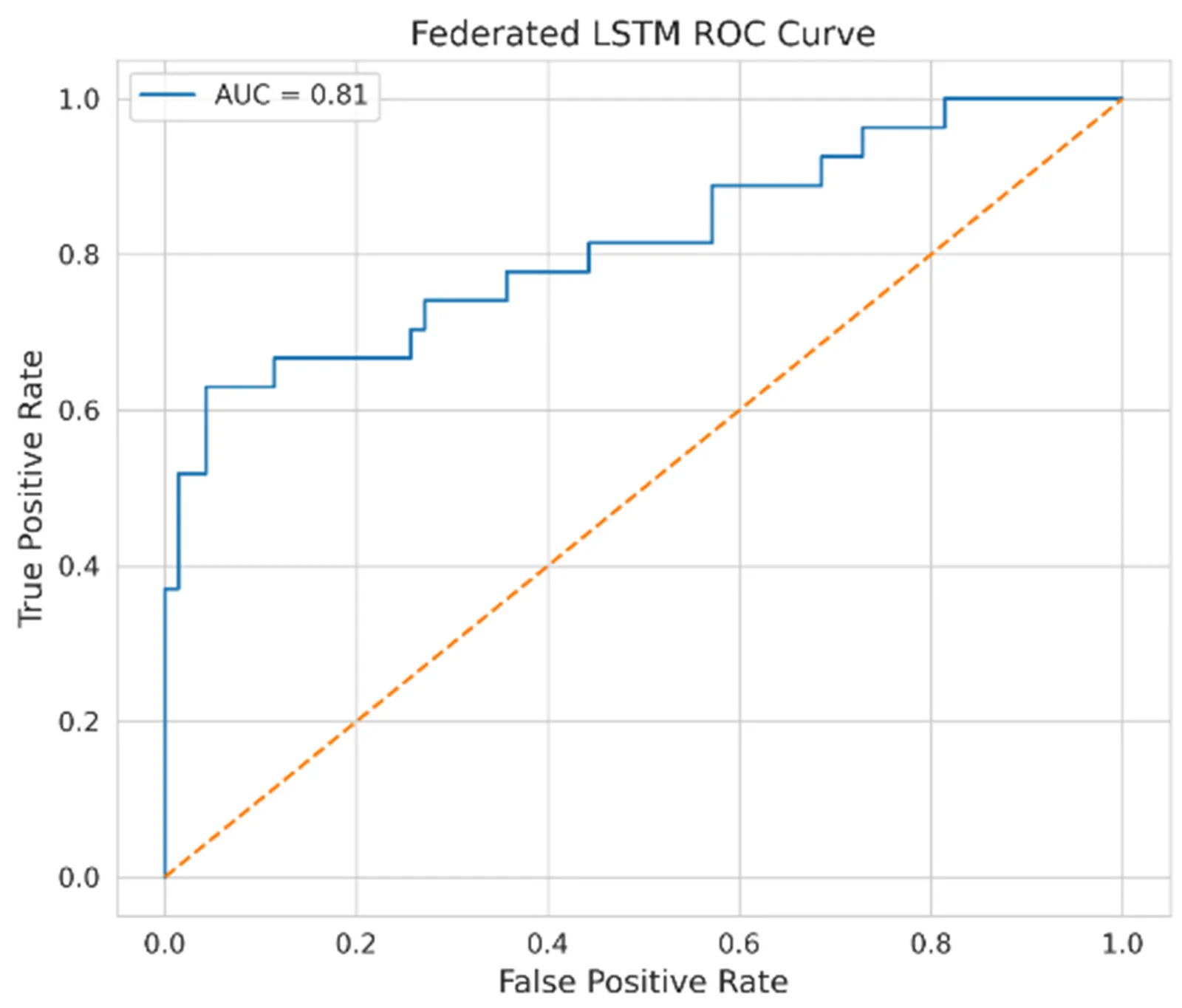
Receiver operating characteristic curve of federated LSTM anomaly detection under stealth cyberattack conditions (AUC = 0.81).

The reduced federated learning performance can be attributed to the presence of non-independent and identically distributed (non-IID) charging behavior across participating clients. Because each charging node observes only localized charging patterns, the global model faces greater difficulty learning generalized anomaly representations compared with centralized learning, where the full operational charging dataset is accessible. The receiver operating characteristic (ROC) curve of the federated LSTM under stealth cyberattack conditions is presented in Figure 15, yielding an area under the curve (AUC) of 0.81.

The federated LSTM achieved an AUC of 0.81 under stealth cyberattacks, indicating reliable discrimination despite the subtle nature of the simulated manipulation and the non-IID distribution of charging behavior across clients. Because the Isolation Forest AUC of 0.86 was obtained under aggressive attacks, the two AUC values are not directly comparable as a measure of model superiority. The difference instead highlights the importance of evaluating anomaly detection methods under consistent attack conditions when making comparative performance claims.

**Figure 16:**
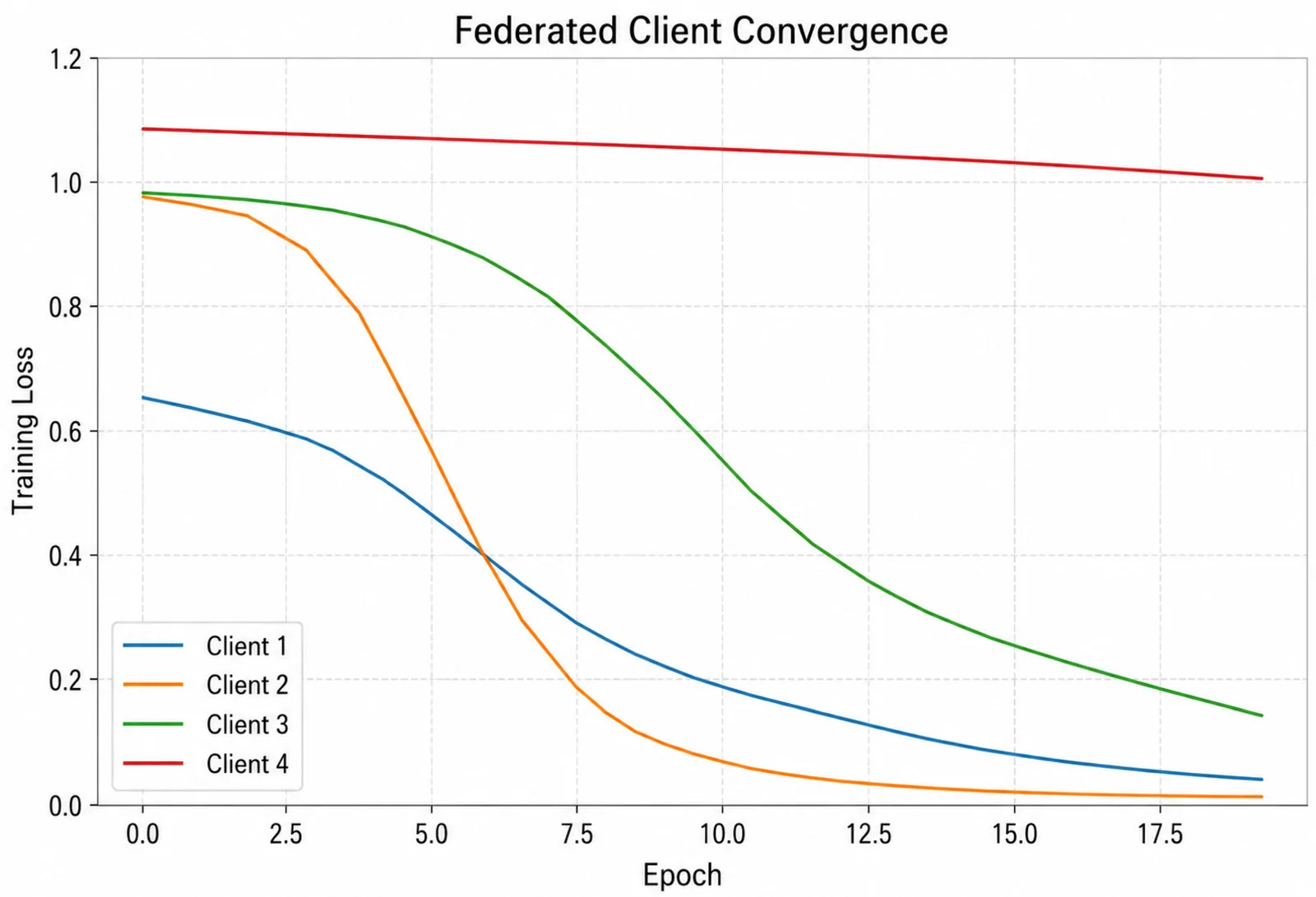
Training convergence behavior of distributed federated clients during decentralized anomaly detection learning.

Figure 16 illustrates the training convergence behavior of the four decentralized federated clients. The clients exhibit different convergence trajectories because each local model is optimized using only its respective local charging distribution. In particular, Client 4 exhibits a comparatively shallow decrease in training loss across the communication rounds. This behavior is consistent with the non-IID nature of the federated setting and does not necessarily indicate a failure of the local optimization process. A heterogeneous client may contain a narrower or more variable representation of charging behavior, including different distributions of charging energy, duration, power, and temporal characteristics. Consequently, the local LSTM autoencoder may require more optimization steps to learn a stable reconstruction representation, resulting in slower loss reduction than clients whose local observations are more internally consistent. Furthermore, the limited number of communication rounds restricts the opportunity for Client 4 to progressively adapt its local representation through repeated global-model initialization and aggregation. The relatively slow convergence of Client 4 therefore provides evidence of the practical optimization challenges associated with non-IID federated EV charging data rather than indicating that the federated framework is unable to train effectively.

Under FedAvg, such client-level differences are incorporated into the global model through weighted parameter aggregation, meaning that a slowly converging client can contribute useful but distribution-specific information without requiring its raw charging data to be shared.

Overall, the findings demonstrate that federated cybersecurity provides a practical balance between privacy preservation and anomaly detection capability. Although centralized learning achieved superior performance, the federated framework maintained effective cyberattack detection while eliminating raw charging data transmission and preserving operational privacy.

### 4.10. ROC-AUC and Comparative Performance Analysis

Receiver Operating Characteristic (ROC) analysis was conducted to evaluate the anomaly discrimination capability of the proposed detection approaches under their respective attack scenarios. For a fair interpretation, AUC values should primarily be compared among models evaluated under the same cyberattack type, because attack severity and statistical separability directly influence anomaly detection performance. In the present study, the Isolation Forest achieved an AUC of 0.86 under aggressive cyberattacks, whereas the federated LSTM achieved an AUC of 0.81 under stealth cyberattacks. These values were obtained under different attack conditions and therefore should not be interpreted as a direct model-to-model comparison. Rather, they demonstrate that both approaches retained meaningful discrimination capability under their respective evaluation scenarios, despite the substantially different attack characteristics.

Isolation Forest achieved an Area Under the Curve (AUC) of 0.86, indicating strong anomaly separability due to substantial operational deviations. In contrast, the federated LSTM achieved an AUC of 0.81 under stealth cyberattacks, demonstrating reliable detection despite subtle manipulation and non-independent and identically distributed (non-IID) charging behavior.

Comparative analysis further showed that aggressive cyberattacks were easier to detect because manipulated charging sessions introduced stronger deviations from normal behavior. Consequently, both statistical and deep learning approaches achieved strong performance, with the centralized LSTM autoencoder demonstrating near-perfect detection capability.

In contrast, stealth cyberattacks increased anomaly detection difficulty due to their similarity to legitimate charging behavior. As summarized in Table 10, Isolation Forest performance degraded under stealth conditions, reducing the attack F1-score to 0.61. Conversely, the centralized LSTM autoencoder maintained stronger detection capability with an attack F1-score of 0.96, demonstrating superior robustness in identifying subtle anomalies through reconstruction-based temporal learning. The federated LSTM achieved an attack F1-score of 0.67, providing meaningful detection capability while preserving distributed data privacy.

**Figure 17:**
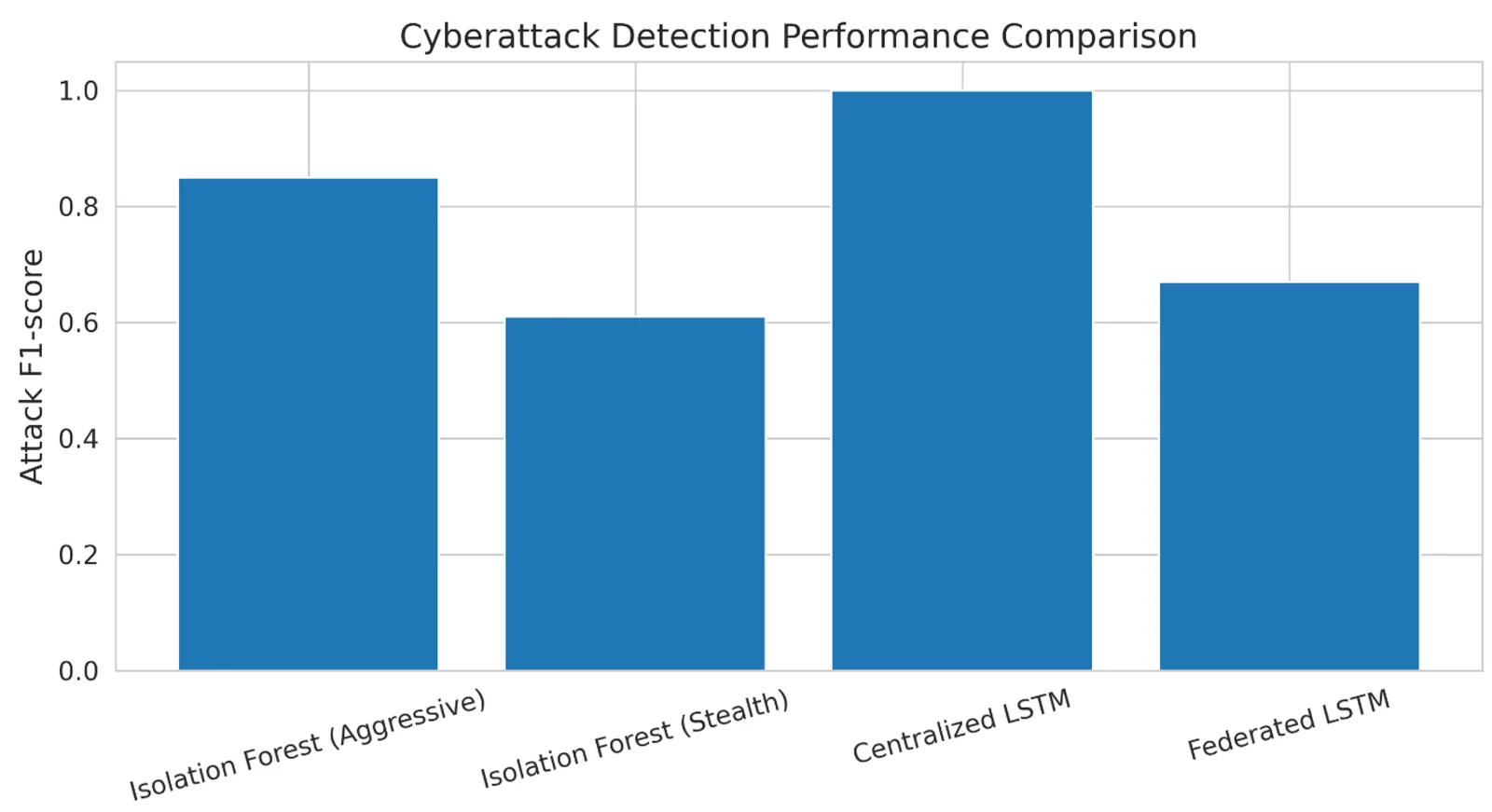
Comparison of attack F1-score performance of anomaly detection frameworks under aggressive and stealth cyberattack conditions.

As illustrated in Figure 17, deep learning-based anomaly detection demonstrated stronger robustness against intelligent charging manipulation that mimics legitimate EV behavior. Although the centralized LSTM achieved superior detection performance, the federated framework provided a privacy-preserving alternative by eliminating raw charging data sharing across charging nodes. This tradeoff between detection performance and privacy preservation represents a key advantage of the proposed framework.

Overall, the results validate the effectiveness of the proposed federated cybersecurity framework for IoT-enabled EV charging infrastructures, demonstrating its capability to detect both aggressive and stealth cyberattacks while preserving distributed operational privacy.

## 5. Discussion

The experimental results highlight the significant impact of cyberattack characteristics on anomaly detection performance in IoT-enabled EV charging infrastructures. Aggressive cyberattacks introduced substantial deviations in charging behavior, resulting in strong separability between legitimate and malicious charging sessions. Consequently, both statistical and deep learning approaches achieved high detection performance. In contrast, stealth cyberattacks introduced only subtle modifications while preserving statistical similarity to normal charging behavior, substantially increasing detection complexity and reducing anomaly separability. The controlled attack injection proportion also supports a consistent comparison across the evaluated attack scenarios. Both aggressive and stealth conditions used 269 synthetic attack samples, corresponding to approximately 14.0% of the original 1929-session dataset, while the detector architectures and training configurations were kept consistent within their respective evaluations. Consequently, differences in detection performance primarily reflect the characteristics and separability of the injected attack behavior rather than changes in attack prevalence or detector configuration.

The comparative evaluation demonstrates that deep learning approaches are more effective than conventional statistical methods for detecting sophisticated cyberattacks. While Isolation Forest achieved satisfactory performance under aggressive attack conditions, its effectiveness decreased considerably under stealth attacks. This performance degradation indicates that traditional anomaly detection methods face challenges when adversarial behavior closely resembles legitimate charging activity. Conversely, the LSTM autoencoder maintained strong detection capability by learning latent temporal representations of charging behavior and identifying subtle inconsistencies through reconstruction error analysis.

A key observation from this study is the tradeoff between centralized learning performance and decentralized data locality. The centralized LSTM autoencoder achieved higher detection performance because it was trained using the complete centralized training distribution, whereas the federated LSTM operated under decentralized learning constraints, with each client accessing only its localized charging data. The federated model achieved lower recall and F1-score under stealth cyberattacks, which can be attributed in part to non-IID charging behavior across participating clients and the resulting difficulty in learning generalized attack representations. Nevertheless, the federated framework avoids direct exchange of raw charging records and therefore reduces direct raw data exposure. This characteristic is relevant to geographically distributed EV charging ecosystems where operational or privacy requirements may restrict centralized collection of charging session data. However, because secure aggregation, differential privacy, and formal leakage-resistance mechanisms were not implemented, the federated approach should not be interpreted as providing a formal privacy guarantee.

The heterogeneous convergence observed in Figure 16 further illustrates the effect of non-IID data on federated optimization. In particular, the comparatively slow reduction in Client 4 training loss indicates that some clients may require more optimization to learn stable representations from their local charging distributions. Although slower local convergence can arise from greater local variability or distributional differences, Client 4 still contributes its learned parameters to the global FedAvg model. Therefore, the slower convergence should be interpreted as a characteristic of decentralized optimization under heterogeneous data rather than solely as a model deficiency.

It should also be noted that the centralized and federated LSTM models used different optimization schedules. The centralized model was trained for 20 epochs, whereas each federated client performed 20 local epochs in each of the four communication rounds. Consequently, the federated configuration involved repeated local optimization and global aggregation and cannot be considered an exactly matched optimization budget with the centralized model. The observed centralized–federated performance difference should therefore be interpreted as reflecting both the effects of decentralized non-IID learning and the respective training protocols. Future work should investigate strictly matched computational and optimization budgets to isolate the contribution of federated learning more precisely.

The difference in evaluation sample size between the centralized and federated experiments should also be considered when interpreting their performance metrics. The centralized stealth evaluation used 1795 samples, whereas the federated global model was evaluated using a fixed test set of 97 charging sessions, including 70 normal and 27 stealth attack samples. Although the fixed federated evaluation set provides a consistent basis for assessing the decentralized model, its relatively small size, particularly the 27 attack samples, introduces greater statistical uncertainty in the estimated attack recall and F1-score. Consequently, the federated F1-score of 0.67 should be interpreted as an empirical estimate under the evaluated test set composition rather than as a definitive measure of generalization performance. Larger and more diverse federated evaluation datasets, together with repeated independent training runs and confidence interval analysis, would provide stronger statistical evidence in future studies.

The scalability of the federated framework also remains an open experimental question. The present study uses four federated clients to demonstrate decentralized anomaly detection under heterogeneous charging conditions; however, four clients are insufficient to establish scalability across a large EV charging ecosystem. In particular, the current experiments do not quantify the statistical severity of client heterogeneity, report comprehensive per-client sample and attack distributions, or measure communication volume and training time. These factors can become increasingly important as the number of participating charging stations grows because client imbalance, stronger distributional divergence, repeated parameter exchange, and local computational constraints may affect both convergence and operational cost. Future work should therefore quantify non-IID severity using formal distributional measures, report client-specific sample counts, attack prevalence, recall, precision, and F1-score, and evaluate communication bytes, local training time, aggregation time, and total training time across progressively larger client populations. Such an evaluation would provide stronger evidence regarding the scalability and practical deployment cost of the proposed federated cybersecurity framework.

The AUC results should also be interpreted with respect to the attack scenario used for evaluation. The Isolation Forest and federated LSTM results reported in Section 4.9 were obtained under aggressive and stealth cyberattack conditions, respectively. Since attack characteristics and anomaly separability differ substantially between these scenarios, direct comparison of the corresponding AUC values is not statistically equivalent to comparing two models under the same attack distribution. Therefore, the reported AUC values are used primarily to demonstrate discrimination capability under their respective attack conditions. A rigorous model-level AUC comparison should evaluate competing methods using the same attack type, test samples, and decision criteria, which is an important direction for future experimental validation.

A key limitation of this study is that the underlying EV charging dataset contains real operational charging records but does not include verified real-world cyberattack events or attack labels. Therefore, the aggressive and stealth cyberattack scenarios were synthetically generated through controlled perturbations of legitimate charging sessions. To improve the realism of the attack modeling, the stealth scenario was grounded in the documented charge manipulation attack (CMA) model reported in [33], and an additional literature-grounded validation was conducted in Section 4.6. The validation provides evidence that the synthetic stealth scenario represents a plausible implementation of a documented EV charging attack mechanism and exhibits the expected characteristics of subtle charging manipulation. Nevertheless, such synthetic attack generation cannot fully reproduce the temporal dependencies, protocol-level characteristics, coordinated behaviors, and adaptive strategies that may occur during actual cyber incidents. Therefore, the results should be interpreted as validation under literature-grounded synthetic attack scenarios rather than direct validation using operational cyberattack traces. Future work should incorporate real attack logs and controlled OCPP-based attack experiments, including laboratory, hardware-in-the-loop, or cyber-range environments, to further validate the proposed framework under operational cybersecurity conditions.

Another limitation concerns the dependence between the engineered cybersecurity-aware features and the synthetic attack generation process. Charging ratio and power consistency are derived from underlying charging variables that are also affected by the attack perturbations. Although these features were recalculated after attack injection to maintain physical and mathematical consistency, this dependency may allow the detectors to exploit feature relationships associated with the specific injection mechanism. Therefore, the present results do not fully establish that the learned anomaly representations are invariant to the attack generation rules. A stronger assessment would require feature-ablation experiments that separately evaluate models with and without the engineered features, together with testing against unseen attack mechanisms and perturbation magnitudes that are not used during attack construction. Such experiments would help determine whether the observed detection performance reflects generalizable attack characteristics rather than recognition of the synthetic injection patterns.

A further limitation concerns the independence of the training and testing data. The present study uses a session-level 80:20 stratified partition, which provides balanced evaluation of legitimate and attack samples but does not enforce chronological separation or entity-level isolation. Consequently, charging sessions originating from the same charging environment or occurring during temporally adjacent operating periods may be represented in both subsets. This configuration can potentially provide the detection models with behavior patterns that are related to the training distribution and may therefore result in more optimistic performance than would be observed when a model is deployed on previously unseen stations, users, or future charging periods. The current dataset does not provide sufficiently persistent user-level identifiers to support a rigorous user held-out evaluation. Future work should therefore investigate chronological hold-out evaluation, charging station held-out testing, and user-level separation where reliable identifiers are available. Such evaluations would provide a stronger assessment of temporal and entity-level generalization and better characterize the deployment robustness of the proposed federated anomaly detection framework.

An important direction for extending the proposed framework is validation using heterogeneous and potentially low-quality real-world charging data collected from geographically distributed charging stations. Operational charging datasets may exhibit substantial heterogeneity due to differences in charger types, charging protocols, user behavior, sampling frequency, measurement accuracy, missing values, communication interruptions, and station-specific operating conditions [34]. Recent work on privacy-preserving collaborative battery fault warning has demonstrated the challenges associated with heterogeneous charging station data and the need for collaborative learning approaches capable of handling differences in data quality and distribution [35]. These characteristics are particularly relevant to the proposed federated anomaly detection framework because non-IID and low-quality local data may further affect model convergence, anomaly representation, and detection reliability. Future work should evaluate the proposed framework using geographically distributed real-world charging datasets containing naturally occurring noise, missing values, class imbalance, and heterogeneous feature distributions, while investigating robust preprocessing, client-adaptive learning, personalized federated learning, and quality-aware aggregation.

The statistical uncertainty and operational evaluation of the reported detection performance represent additional limitations of the present study. The reported attack F1-score and ROC-AUC values, including 0.85 and 0.86 for Isolation Forest under aggressive attacks and 0.67 and 0.81 for the federated LSTM under stealth attacks, were obtained from the implemented experimental configuration and are not accompanied by confidence intervals across independent random seeds or alternative federated client partitions. Therefore, these values should be interpreted as point estimates for the evaluated configuration rather than as estimates of performance variability across repeated experimental conditions. In addition, the present evaluation focuses on accuracy, precision, recall, F1-score, and ROC-AUC and does not include precision–recall area under the curve (PR-AUC), false alarms normalized by charger day, detection delay, or recall reported separately for each attack mechanism. These metrics are particularly important for practical EV charging cybersecurity because attack prevalence, false alarm burden, and the time required to identify malicious charging behavior directly affect operational usability. Future work should therefore perform repeated experiments across multiple random seeds and federated client partitions and report confidence intervals for the principal detection metrics. The evaluation should additionally include PR-AUC, false alarms per charger day, detection delay, and per-attack recall to provide a more comprehensive assessment of statistical robustness and operational cybersecurity performance.

Several additional operational and adversarial conditions remain outside the scope of the present evaluation. In particular, the current experiments do not explicitly evaluate concept drift caused by evolving charging behavior, charger outages or communication failures, benign anomalies arising from legitimate but unusual charging activity, or poisoning attacks targeting federated model updates. These conditions are important for practical deployment because an anomaly detector may encounter distributional changes and operational abnormalities that are not necessarily malicious, while compromised federated clients may intentionally submit manipulated model updates. Future work should therefore evaluate the proposed framework under temporally evolving charging distributions, charger outages and communication interruptions, naturally occurring benign anomalies, and malicious model update scenarios. Robust federated aggregation methods should also be investigated to improve resilience against poisoned updates and compromised clients. Such evaluations would provide a more comprehensive assessment of operational robustness and distinguish genuine cyberattacks from legitimate abnormal charging behavior.

Overall, the results demonstrate the feasibility of federated anomaly detection for EV charging cybersecurity under the evaluated aggressive and stealth synthetic attack scenarios while avoiding direct exchange of raw charging records. However, the current study does not establish robustness against concept drift, charger outages, benign operational anomalies, poisoned federated updates, or other adaptive adversarial conditions, nor does it provide formal privacy guarantees through mechanisms such as secure aggregation or differential privacy. Therefore, the findings should be interpreted as evidence of anomaly detection capability under controlled attack conditions rather than as validation of a fully secure or privacy-preserving operational deployment. Future work should integrate realistic OCPP-based attack scenarios, concept drift adaptation, outage-aware detection, benign anomaly discrimination, robust federated aggregation, secure aggregation, and differential privacy to establish stronger cybersecurity, robustness, and privacy guarantees for real-world EV charging infrastructures.

## 6. Conclusions

This paper proposed a privacy-preserving federated cybersecurity framework for IoT-based electric vehicle (EV) charging infrastructures using distributed anomaly detection. The framework integrates cybersecurity-aware feature engineering, cyberattack modeling, statistical and deep learning-based anomaly detection, and federated learning to improve cybersecurity resilience while preserving charging privacy.

Experimental evaluation using a real-world EV charging dataset under aggressive and stealth cyberattack scenarios demonstrated that aggressive attacks were easier to detect due to stronger operational deviations, whereas stealth attacks significantly increased detection difficulty because of their similarity to legitimate charging behavior. Comparative results showed that deep learning approaches, particularly the centralized LSTM autoencoder, achieved superior anomaly detection performance under stealth attacks. Although the federated LSTM framework showed lower performance than centralized learning, it maintained competitive detection capability while enabling privacy-preserving collaborative learning without transmitting raw charging data.

Future work will investigate geographically distributed charging stations, multi-source datasets, real-time Open Charge Point Protocol (OCPP) communication streams, and secure aggregation mechanisms. Advanced anomaly detection techniques, including transformer-based models, graph neural networks, and adaptive threat intelligence, may also be explored to improve cybersecurity scalability and robustness in smart charging environments.

## Figures and Tables

**Table 1:** The representative literature related to EV charging cybersecurity, anomaly detection, IOT intrusion detection, and federated learning while highlighting the research gap addressed by the proposed framework.

Ref.	Research Focus	Methodology	Cyberattack Scenario	Federated Learning	Major Limitation
[27]	EV charging anomaly detection	ML-based anomaly detection	False data injection	No	Centralized architecture with privacy concerns
[28]	Smart charging cybersecurity	Deep learning intrusion detection	Charging manipulation	No	No privacy-preserving learning mechanism
[29]	IoT-enabled charging security	LSTM-based anomaly detection	Session spoofing	No	Centralized training and limited scalability
[30]	Federated IoT cybersecurity	Federated neural networks	General cyberattacks	Yes	Not EV charging specific
[31]	EV charging operational analytics	Charging behavior analysis	Not considered	No	No cybersecurity evaluation
[32]	Smart grid cybersecurity	Federated anomaly detection	False data injection	Yes	Limited applicability to EV charging environments
Proposed Work	IoT-based EV charging cybersecurity	Isolation Forest + Centralized LSTM + Federated LSTM	Aggressive + Stealth cyberattacks	Yes	Privacy-preserving distributed anomaly detection with realistic attack modeling

**Table 2:** Descriptive statistics of EV charging features.

Feature	Mean	Std. Dev.	Min	25%	Median	75%	Max
Usage (kWh)	35.55	26.61	0.50	17.10	29.80	47.10	229.69
Duration (Hours)	3.24	1.87	0.08	1.98	3.10	4.15	13.60
Average Output (kW)	9.26	5.38	1.00	5.00	10.00	11.00	49.25
Charging Ratio	16.18	22.36	1.44	6.46	10.79	11.45	208.18
Power Consistency	6.97	18.89	0.00	0.33	0.48	0.81	180.35

**Table 3:** Aggressive cyberattack injection configuration.

Parameter	Configuration
Attack type	Aggressive charging manipulation
Attack samples	269
Attack injection proportion	≈14.0% of 1929 sessions
Manipulated variables	Energy, duration, power
Energy equation	$\left(E^{\left(a\right)}=E\left(1+\alpha \right)\right)$
Duration equation	$\left(D^{\left(a\right)}=D\left(1+\beta \right)\right)$
Perturbation distribution	U (−0.10, 0.10)
Power calculation	$\left(P^{\left(a\right)}=E^{\left(a\right)}/D^{\left(a\right)}\right)$
Physical constraints	$\left(E^{\left(a\right)}>0,;D^{\left(a\right)}>0,;P^{\left(a\right)}\right)$ within observed operational bounds
Timestamp handling	Original charging timestamps retained; temporal features were derived from the original timestamps
SOC	Not available; not directly modeled
Derived features	Recalculated after manipulation
Attack objective	Highly disruptive charging manipulation

**Table 4:** Isolation Forest performance under aggressive cyberattacks.

Accuracy	Precision	Recall	F1-Score	AUC
**0.96**	0.84	0.87	0.85	0.86

**Table 5:** Centralized LSTM performance under aggressive cyberattacks.

Accuracy	Precision	Recall	F1-Score
1.00	1.00	1.00	1.00

**Table 6:** Stealth cyberattack injection summary.

Parameter	Configuration
Attack type	Stealth charge manipulation
Attack samples	269
Attack injection proportion	≈14.0% of 1929 sessions
Manipulated variables	Energy, duration
Energy perturbation	$\left(E^{\left(s\right)}=E\left(1+\epsilon_{E}\right)\right)$
Duration perturbation	$\left(D^{\left(s\right)}=D\left(1+\epsilon_{D}\right)\right)$
Perturbation distribution	$\left(\epsilon_{E},\epsilon_{D}∼U\left(-0.10,0.10\right)\right)$
Resulting power	$\left(P^{\left(s\right)}=E^{\left(s\right)}/D^{\left(s\right)}\right)$
Physical constraints	Positive duration and energy; power constrained to operational range
Timestamp handling	Original charging timestamps retained; temporal features were derived from the original timestamps
SOC	Not available; not directly modeled
Derived features	Recalculated after manipulation
Attack objective	Subtle/undetected charging manipulation
Detection difficulty	High

**Table 7:** Isolation Forest performance under stealth cyberattacks.

Accuracy	Precision	Recall	F1-Score
0.88	0.59	0.63	0.61

**Table 8:** Centralized LSTM autoencoder performance under stealth cyberattacks.

Class	Precision	Recall	F1-Score	Support
Normal (0)	0.99	0.99	0.99	1526
Attack (1)	0.96	0.95	0.96	269
Accuracy	—	—	0.99	1795
Macro Average	0.98	0.97	0.97	1795
Weighted Average	0.99	0.99	0.99	1795

**Table 9:** Federated LSTM performance under stealth cyberattacks.

Class	Precision	Recall	F1-Score	Support
Normal (0)	0.84	0.99	0.91	70
Attack (1)	0.93	0.52	0.67	27
Accuracy	—	—	0.86	97
Macro Average	0.89	0.75	0.79	97
Weighted Average	0.87	0.86	0.84	97

**Table 10:** Comparison of cyberattack detection.

Model	Accuracy	Attack F1-Score	AUC	Privacy-Preserving
Isolation Forest (Aggressive)	0.96	0.85	0.86	No
Isolation Forest (Stealth)	0.88	0.61	—	No
Centralized LSTM (Stealth)	0.99	0.96	—	No
Federated LSTM (Stealth)	0.86	0.67	0.81	Yes

## Data Availability

The EV charging dataset used in this study consists of real-world charging session records collected from an IoT-enabled electric vehicle charging infrastructure. Due to data ownership, privacy, and operational confidentiality restrictions, the dataset is not publicly available. However, anonymized data samples and the source code used for data preprocessing, cyberattack simulation, anomaly detection, and federated learning experiments may be made available by the corresponding author upon reasonable request and subject to approval from the data provider.

## References

[1] Aldhanhani T., Abraham A., Hamidouche W., Shaaban M. (2024). Future Trends in Smart Green IoV: Vehicle-to-Everything in the Era of Electric Vehicles. IEEE Open J. Veh. Technol..

[2] Karim S., Chauhdary S.T., Kamil H., Mumtaz F., Farid G. (2026). Wireless Power Transfer and IoT in EV Charging: Opportunities, Challenges, and Future Directions. IEEE Access.

[3] Dehrouyeh F., Yang L., Badrkhani Ajaei F., Shami A. (2024). On TinyML and Cybersecurity: Electric Vehicle Charging Infrastructure Use Case. IEEE Access.

[4] Zografopoulos I., Hatziargyriou N.D., Konstantinou C. (2023). Distributed Energy Resources Cybersecurity Outlook: Vulnerabilities, Attacks, Impacts, and Mitigations. IEEE Syst. J..

[5] Poorani S., Sivaraju S.S., Arthy G., Manjusha M. (2025). Advancing machine learning-driven cybersecurity solutions for secure electric vehicle charging in smart grids. Int. J. Inf. Technol..

[6] Maleki S., Pan S., Lakshminarayana S., Konstantinou C. (2025). Survey of Load-Altering Attacks Against Power Grids: Attack Impact, Detection, and Mitigation. IEEE Open Access J. Power Energy.

[7] Elshazly A.A., Elgarhy I., Eltoukhy A.T., Mahmoud M., Eberle W., Alsabaan M., Alshawi T. (2024). False Data Injection Attacks on Reinforcement Learning-Based Charging Coordination in Smart Grids and a Countermeasure. Appl. Sci..

[8] Tanyıldız H., Şahin C.B., Dinler Ö.B., Migdady H., Saleem K., Smerat A., Gandomi A.H., Abualigah L. (2025). Detection of cyber attacks in electric vehicle charging systems using a remaining useful life generative adversarial network. Sci. Rep..

[9] Hossen M.S., Sarker M.T., Nabi M.S., Bannah H., Ramasamy G., Eng Eng N. (2025). Federated AI-OCPP Framework for Secure and Scalable EV Charging in Smart Cities. Urban Sci..

[10] Hossen M.S., Ramasamy G., Sarker M.T., Eng N.E. (2026). Real-World Tariff-Aware Safe Reinforcement Learning for Grid-Stable OCPP EV Charging Networks. IEEE Access.

[11] Li S., Yuan S., Guo S., Yu J., Pei X., Zhang Z. (2026). Collaborative Optimization Scheduling Strategy of Electric Vehicle Load Cluster and Data Center for Multi-Station Integration. IEEE Access.

[12] Hijgenaar S., Ştefanov A., Voorden A.M.V., Palensky P. (2025). Cyber Resilience of Electric Vehicle Charging in Smart Grids: The Dutch Case. IEEE Access.

[13] Yuvaraj T., Devabalaji K.R., Kumar J.A., Thanikanti S.B., Nwulu N.I. (2024). A Comprehensive Review and Analysis of the Allocation of Electric Vehicle Charging Stations in Distribution Networks. IEEE Access.

[14] Ali Khan M., Rais R.N.B., Khalid O., Deriche M. (2025). Comparative Analysis of Centralized and Federated Intrusion Detection in IoT-Enabled Cyber-Physical Systems Under Data and Label-Skew. IEEE Access.

[15] Ali A., Jianjun H., Jabbar A. (2025). Recent Advances in Federated Learning for Connected Autonomous Vehicles: Addressing Privacy, Performance, and Scalability Challenges. IEEE Access.

[16] Liu Y., Liu Y., Zhang W., Luo D., Qiao Q., Cao S., Zhang B., Chen T., Zhao H., Li X. (2026). Eliminate Conflicts and Attacks: Fair and Robust Federated Learning for Anomaly Detection of Charging Stations. IEEE Trans. Intell. Transp. Syst..

[17] AlHousrya O., Bennagi A., Cotfas P.A., Cotfas D.T. (2025). The Role of the Industrial IoT in Advancing Electric Vehicle Technology: A Review. Appl. Sci..

[18] Sepehrzad R., Faraji M.J., Al-Durra A., Sadabadi M.S. (2024). Enhancing Cyber-Resilience in Electric Vehicle Charging Stations: A Multi-Agent Deep Reinforcement Learning Approach. IEEE Trans. Intell. Transp. Syst..

[19] Asit Kumar Majhi A., Mohanty S. (2024). A Comprehensive Review on Internet of Things Applications in Power Systems. IEEE Internet Things J..

[20] Zhang H., Niyato D., Zhang W., Zhao C., Du H., Jamalipour A., Sun S., Pei Y. (2025). The Role of Generative Artificial Intelligence in Internet of Electric Vehicles. IEEE Internet Things J..

[21] Hossen M.S., Ramasamy G., Al Qwaid M. (2026). An Integrated Machine Learning Framework for EV Charging Behavior Characterization and Anomaly Detection in Public Charging Infrastructure. Appl. Sci..

[22] Khan J.A., Khan M.A., Saeed N., Cayrel P.-L., Hahn C. (2025). Intrusion Detection Systems for In-Vehicle Networks: Protocols, Applications, and Challenges. IEEE Access.

[23] Quan M.K., Pathirana P.N., Wijayasundara M., Setunge S., Nguyen D.C., Brinton C.G., Love D.J., Poor H.V. (2026). Federated Learning for Cyber Physical Systems: A Comprehensive Survey. IEEE Commun. Surv. Tutor..

[24] Khan N., Nisar S., Khan M.A., Ur Rehman Y.A., Noor F., Barb G. (2025). Optimizing Federated Learning With Aggregation Strategies: A Comprehensive Survey. IEEE Open J. Comput. Soc..

[25] Hamad N.A., Bakar K.A.A., Qamar F., Jubair A.M., Mohamed R.R., Mohamed M.A. (2025). Systematic Analysis of Federated Learning Approaches for Intrusion Detection in the Internet of Things Environment. IEEE Access.

[26] Amanlou S., Hasan M.K., Mokhtar U.A., Malik K.M., Islam S., Khan S., Khan M.A. (2025). Cybersecurity Challenges in Smart Grid Systems: Current and Emerging Attacks, Opportunities, and Recommendations. IEEE Open J. Commun. Soc..

[27] Mitikiri S.B., Tiwari Y., Srinivas V.L., Pal M. (2025). Regression based anomaly detection in electric vehicle state of charge fluctuations through analysis of electric vehicle charging infrastructure data. Sustain. Energy Grids Netw..

[28] Jahangir H., Lakshminarayana S., Poor H.V. (2024). Charge Manipulation Attacks Against Smart Electric Vehicle Charging Stations and Deep Learning-Based Detection Mechanisms. IEEE Trans. Smart Grid.

[29] Hossen M.S., Sarker M.T., Al Qwaid M., Ramasamy G., Eng Eng N. (2025). AI-Driven Framework for Secure and Efficient Load Management in Multi-Station EV Charging Networks. World Electr. Veh. J..

[30] Hossen M.S., Ramasamy G., Eng Eng N., Qwaid M.A. (2026). An Anomaly-Aware, Q-Learning Framework for Real-Time Scheduling in Multi-Station EV Charging Networks. Electronics.

[31] Wang S., Liu B., Li Q., Han D., Zhou J., Xiang Y. (2025). EV Charging Behavior Analysis and Load Prediction via Order Data of Charging Stations. Sustainability.

[32] Hossen M.S., Ramasamy G., Eng N.E. (2026). AI-OCPP Framework for Smart and Safe EV Charging: Real-World Performance Evaluation. IEEE Access.

[33] Xiao Q., Song L., Woo J.H., Hu R., Xu B., Ye K., Lu N. (2025). Invisible Manipulation: Deep Reinforcement Learning-Enhanced Stealthy Attacks on Battery Energy Management Systems. IEEE Access.

[34] Aghsaee R., Hecht C., Schwinger F., Figgener J., Jarke M., Sauer D.U. (2023). Data-Driven, Short-Term Prediction of Charging Station Occupation. Electricity.

[35] Yang H., Tian J., Mai W., Wang C., Ran L., Wu T., He S., Wang Z., Shi X., Liang Z. (2026). Privacy-preserving collaborative battery fault warning for massive electric vehicles by heterogeneous data from charging stations. Nat. Commun..

